# The role of a ciliary GTPase in the regulation of neuronal maturation of olfactory sensory neurons

**DOI:** 10.1242/dev.201116

**Published:** 2023-01-19

**Authors:** Julien C. Habif, Chao Xie, Carlos de Celis, Kirill Ukhanov, Warren W. Green, Jordan C. Moretta, Lian Zhang, Robert J. Campbell, Jeffrey R. Martens

**Affiliations:** ^1^ Department of Pharmacology and Therapeutics, University of Florida, College of Medicine, Gainesville, FL 32610, USA; ^2^ University of Florida Center for Smell and Taste, Gainesville, FL 32610, USA

**Keywords:** ARL13B, Maturation, Ciliopathies, Primary cilia, Olfactory sensory neurons, Olfaction, Mouse

## Abstract

Olfactory sensory neurons (OSNs) form embryonically and mature perinatally, innervating glomeruli and extending dendrites with multiple cilia. This process and its timing are crucial for odor detection and perception and continues throughout life. In the olfactory epithelium (OE), differentiated OSNs proceed from an immature (iOSN) to a mature (mOSN) state through well-defined sequential morphological and molecular transitions, but the precise mechanisms controlling OSN maturation remain largely unknown. We have identified that a GTPase, ARL13B, has a transient and maturation state-dependent expression in OSNs marking the emergence of a primary cilium. Utilizing an iOSN-specific *Arl13b*-null murine model, we examined the role of ARL13B in the maturation of OSNs. The loss of *Arl13b* in iOSNs caused a profound dysregulation of the cellular homeostasis and development of the OE. Importantly, *Arl13b* null OSNs demonstrated a delay in the timing of their maturation. Finally, the loss of *Arl13b* resulted in severe deformation in the structure and innervation of glomeruli. Our findings demonstrate a previously unknown role of ARL13B in the maturation of OSNs and development of the OE.

## Teaser

**Summary:** ARL13B, a protein enriched in a signaling hub (cilia), is required for the maturation of neurons in the mouse nose and its loss impairs smell.

## INTRODUCTION

The olfactory epithelium (OE) is in direct contact with the external environment rendering it highly susceptible to injury. Importantly, as a neuroprotective measure the OE undergoes life-long constitutive regeneration. The continuous repopulation of the OE is facilitated by the stem cells, globose basal cells (GBCs), that give rise to every cell type in the OE (Schwob et al., 2017). Within their neuronal lineage, GBCs differentiate into immature olfactory sensory neurons (iOSNs), which bidirectionally extend their dendrite towards the nasal cavity and their axon towards the olfactory bulb (OB). iOSNs then proceed to become mature OSNs (mOSNs), which undergo multi-ciliogenesis and establish synaptic connections in glomeruli in the OB (Liberia et al., 2019; Rodriguez-Gil et al., 2015). Although tremendous effort has been placed in detailing the timing of crucial biological processes during the maturation of OSNs (Kondo et al., 2010; Nickell et al., 2012; Rodriguez-Gil et al., 2015; Coleman et al., 2017; Fletcher et al., 2017; Liberia et al., 2019; McClintock et al., 2020; Wang et al., 2017; Kurian et al., 2021; Dorrego-Rivas and Grubb, 2022), little is understood about the signaling mechanisms responsible for their maturation. Interestingly, there is evidence that neuronal primary cilia, known as ‘signaling antennas’, play crucial roles in the formation and maturation of neurons (Youn and Han, 2018; Han et al., 2008) and the regulation of the early patterning of another neuronal tissue, the neural tube (Caspary et al., 2007). Within the OE, primary cilia are present in a reserve population of stem cells, horizontal basal cells (HBCs); however, these cilia mediate the regeneration of the OE after massive injury and have no impact on its homeostasis (Joiner et al., 2015). Interestingly, an early electron microscopy study detailing ciliogenesis discovered that OSNs possess a ‘presumptive’ primary cilium distinct from the fully elongated cilia during OE development (Menco and Farbman, 1985b). This primary cilium identified on OSNs may have a similar developmental role as primary cilia on other neuronal cell types. Therefore, we hypothesized that the primary cilium on OSNs is important for neuronal maturation.

Most primary cilia express the small regulatory GTPase ARL13B, leading to its use as a canonical marker for primary cilia (Delling et al., 2013; Bangs et al., 2015; Li et al., 2021). *ARL13B* is a causative gene for the ciliopathy Joubert syndrome (JS), a multi-system hereditary disorder in humans that results in defects in primary cilia (Cantagrel et al., 2008). ARL13B is involved in various cellular processes, including cilia biogenesis and maintenance (Cantagrel et al., 2008; Higginbotham et al., 2012), axon guidance (Ferent et al., 2019) and the Hedgehog (HH) signaling pathway (Caspary et al., 2007; Gigante et al., 2020; Larkins et al., 2011; Mariani et al., 2016; Su et al., 2012). Even with a plethora of studies, the role of ARL13B in the olfactory system was unknown.

Surprisingly, in this study we identify the presence of an ARL13B-positive primary cilium on iOSNs and do not detect ARL13B in the multiple, long cilia of mOSNs. Loss of *Arl13b* in iOSNs resulted in a delay in neuronal maturation and an inability for the OE to form properly. Additionally, loss of *Arl13b* resulted in the deformation of glomerular shape and an attenuation of its innervation. Overall, this study demonstrates for the first time that ARL13B in iOSNs regulates the maturation of neurons in the OE.

## RESULTS

### iOSNs possess ARL13B-positive primary cilia

**Fig. 1.**
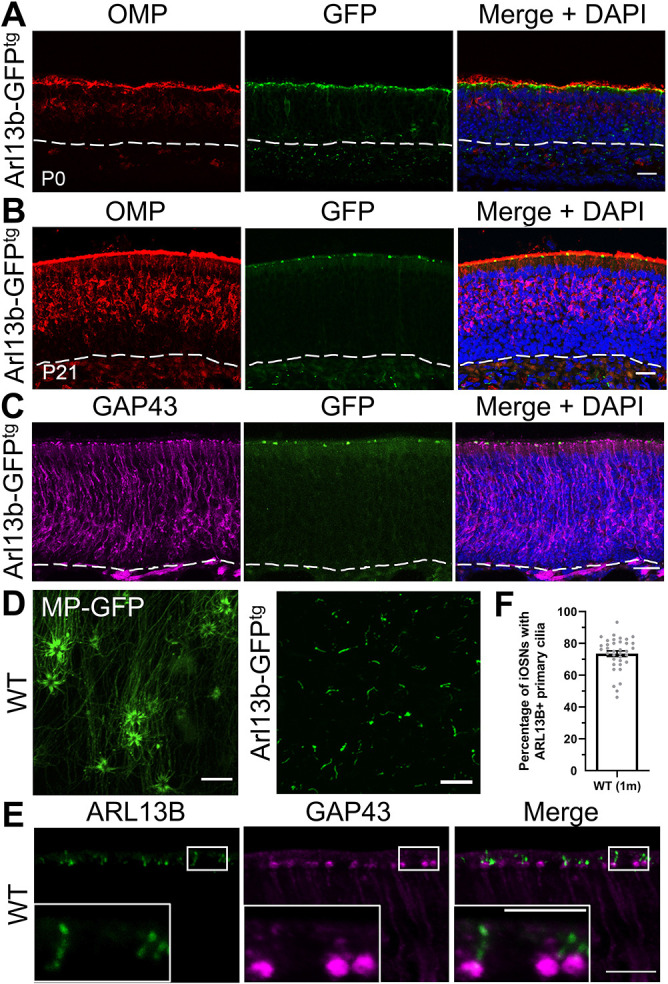
**iOSNs possess primary cilia that express ARL13B.** (A,B) Representative confocal images of the nasal cavity of *Arl13b-GFP^tg^* mice at P0 (A) and P21 (B) immunolabeled with OMP. (C) Representative confocal image of the nasal cavity of *Arl13b-GFP^tg^* mice at P21 immunolabeled with GAP43. (D) *En face* confocal image of the cilia of mOSNs transduced with MP-GFP (left) and the ARL13B-GFP signal of *Arl13b-GFP^tg^* mice (right). (E) Higher magnification image of endogenous immunolabeling of ARL13B in 1-month-old WT mice. (F) Quantification of the percentage of iOSNs with ARL13B^+^ cilia in 1-month-old mice (73.55±1.762%, *n*=4 mice). Dashed lines delineate basement membrane. Scale bars: 5 μm (E insets); 10 μm (D, E main panels); 20 μm (A-C). Images are representative of ten samples per mouse analyzed. Values represent mean±s.e.m.

Early electron microscopy studies of the developing OE identified what was described as a ‘presumptive’ primary cilium in neurons (Menco and Farbman, 1985a; Menco and Farbman, 1985b). However, it remained unclear whether neuronal primary cilia were also present in the OE of postnatal mice. To visualize primary cilia, a reporter mouse model was used in which ARL13B, a common marker of cilia, was fused with a fluorescent tag (*Arl13b-GFP^tg^*) (Delling et al., 2013). We first demonstrated that the *Arl13b-GFP^tg^* mouse is a reliable reporter in the OE, by recapitulating a previous finding (Joiner et al., 2015) of ARL13B-positive, primary cilia emanating from the olfactory progenitors, HBCs, which are marked by keratin 5 (K5; KRT5) (Fig. S1A). To understand the expression pattern of ARL13B in the OE, *Arl13b-GFP^tg^* mice at postnatal day (P) 0 and P21 were immunolabeled for olfactory marker protein (OMP), a marker of mOSNs, and the ARL13B-GFP signal was visualized. The apical surface of the OE, where OSN dendrites reside, showed a discrete, punctate distribution of ARL13B-positive cilia in the *Arl13b-GFP^tg^* mice at both P0 (Fig. 1A) and P21 (Fig. 1B). Notably, the density of ARL13B-GFP signal at the apical surface decreased in P21 mice compared with P0 mice as the OE matured, evidenced by its increase in thickness and number of mOSNs. Interestingly, unlike in the OE, in the adjacent respiratory epithelium (RE) there was robust ARL13B-GFP signal where the multi-cilia of the epithelial cells reside (Fig. S1B). Additionally, at P21 the punctate pattern of ARL13B-GFP labeling in the OE strongly contrasted the robust labeling of the cilia layer of mOSN visualized as a dense band of OMP staining (Fig. 1B). ​When merged, the ARL13B-GFP signal appeared to reside below the layer of the OMP-positive, mature cilia. The physical separation and distinct expression pattern indicated that ARL13B was not detected in the cilia of mOSNs of *Arl13b-GFP^tg^* mice. Therefore, ​to investigate which cell type expressed ARL13B, coronal sections of the OE of P21 aged *Arl13b-GFP^tg^* mice were stained for the iOSN marker growth associated protein 43 (GAP43). The ARL13B-GFP signal colocalized with the dendritic knobs of iOSNs (Fig. 1C). To resolve the ARL13B signal further, we utilized *en face* imaging in a live preparation of the OE with synaptic connections in the OB maintained. *En face* imaging of the surface of the OE of the *Arl13b-GFP^tg^* mouse (Fig. 1D, right) showed short (∼1-5 µm), sparsely located primary cilia. This was a clear contrast in length and number of the primary cilia compared with the dense meshwork of cilia of mOSNs, which were labeled by ectopic expression of the fluorescent ciliary tag adenovirus 5-myristoylated-palmitoylated-GFP (AV5-MP-GFP) (Fig. 1D, left). It is important to note that mOSNs are the only neurons in the OE infected by AV5, as shown by previous work in the laboratory (McIntyre et al., 2012). To examine endogenous expression of ARL13B, we immunostained coronal sections of OE of 1-month-old mice with antibodies against ARL13B and GAP43. Representative high-magnification images of endogenous ARL13B in a wild-type (WT) mouse showed the localization of ARL13B in solitary rod-like projections extending from the dendritic knobs of iOSNs (Fig. 1E), corroborating the *Arl13b-GFP^tg^* mouse data. We determined that about 73% of iOSNs possess an ARL13B-positive primary cilium in 1-month-old WT mice (Fig. 1F). Together, these findings show that iOSNs possess a primary cilium that is ARL13B positive, whereas the multi-cilia of mOSNs lack ARL13B expression.

### Genetic deletion of *Arl13b* under the *123* promoter results in loss of ARL13B in iOSNs

**Fig. 2.**
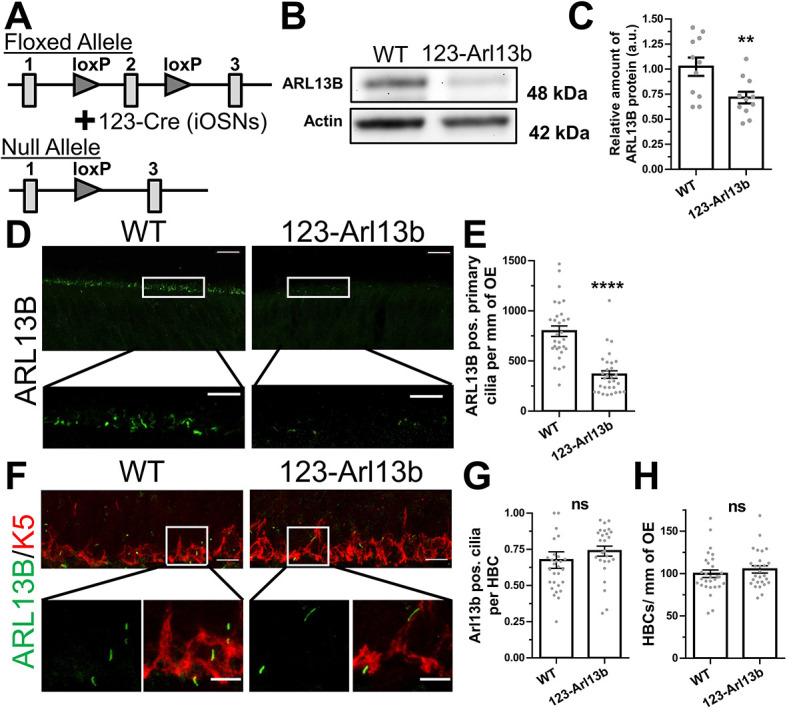
**Genetic deletion of *Arl13b* under the *123* promoter results in loss of ARL13B in iOSNs.** (A) Schematic of the conditional *Arl13b* knockout murine model. Boxes represent exons, triangles loxP insertions. (B) Representative immunoblot image of olfactory mucosa lysates from *123-Arl13b* and WT mice at 1 month of age, probed against ARL13B (48 kDa) and actin (42 kDa). (C) Quantification of the relative amount of ARL13B immunolabeling normalized to actin (WT, 1.024±0.091 a.u., *n*=11 mice; *123-Arl13b*, 0.716±0.058 a.u., *n*=11 mice). (D) Representative images of immunostaining of ARL13B at the apical surface of the OE in P5 mice. (E) Quantification of the number of ARL13B^+^ cilia at the apical surface (WT, 797.3±52.35 cilia, *n*=3 mice; *123-Arl13b*, 364.4±37.89 cilia, *n*=3 mice). (F) Representative images of immunostaining of ARL13B and K5 at the base of the OE in WT and *123-Arl13b* mice at P5. (G) Quantification of ARL13B^+^ cilia on HBCs (WT, 0.6755±0.05696 cilia, *n*=3 mice; *123-Arl13b*, 0.7376±0.03453 cilia, *n*=3 mice). (H) Quantification of the number of HBCs per mm (WT, 99.70±4.464 HBCs/mm, *n*=3 mice; *123-Arl13b*, 104.9±4.264 HBCs/mm, *n*=3 mice). Scale bars: 5 μm (insets); 10 μm (main panels). Images are representative of ten samples per mouse analyzed. **P<0.01 and ****P<0.0001 (unpaired, two-tailed Student's *t*-tests). Values represent mean±s.e.m. a.u., arbitrary units; ns, not significant (*P*>0.05).

Next, we generated an *Arl13b* cKO mouse line by crossing *Arl13b^fl/fl^* mice (Su et al., 2012) with *123-Cre* mice (Hirata et al., 2006; Kaneko-Goto et al., 2013) (Fig. 2A). For the following study, the WT mice were genotypically *123-Cre^+/+^;Arl13b^fl/fl^* and the cKO mice referred to as *123-Arl13b* mice were *123-Cre^+/−^;Arl13b*^Δ/Δ^. We validated the loss of ARL13B protein expression using whole lysate immunoblotting of the olfactory mucosa of 1-month-old mice. Analysis of the immunoblots revealed a decrease in the relative amount of ARL13B in *123-Arl13b* mice compared with their WT littermates (Fig. 2B,C). The reduction and incomplete loss of ARL13B protein in the conditional mouse model was expected because the olfactory mucosa preparation includes various other cell types that possess ARL13B expressing cilia, such as HBCs (Joiner et al., 2015; Fig. S1A), respiratory columnar cells (Fig. S1B), and several cell types in the lamina propria (McClintock et al., 2020). We then assessed the immunoreactivity of ARL13B at P5, an age at which the number of iOSNs is equivalent in WT and *123-Arl13b* mice (Fig. S3A,B). There was a decrease in the number of ARL13B-positive cilia in *123-Arl13b* mice compared with WT (Fig. 2D,E). Possible explanations for the remaining ARL13B-positive cilia at the apical surface of the OE in *123-Arl13b* mice could be: (1) onset of *Arl13b* expression precedes that of *123-Cre*, (2) the relative stability of ARL13B protein (Roy et al., 2017) and/or (3) the short time frame of OSN immaturity (Rodriguez-Gil et al., 2015; Liberia et al., 2019) with the continual renewal of iOSNs. Finally, the other ciliated cell type, HBCs, in the OE showed no change in their number of ARL13B-positive cilia (Fig. 2F-H), indicating the specificity of ARL13B loss in iOSNs of *123-Arl13b* mice. The demonstrated conditional loss of ARL13B protein in iOSNs provides a model to assay the role of ARL13B in the development of the OE.

### Loss of ARL13B in iOSNs leads to improper OE development

**Fig. 3.**
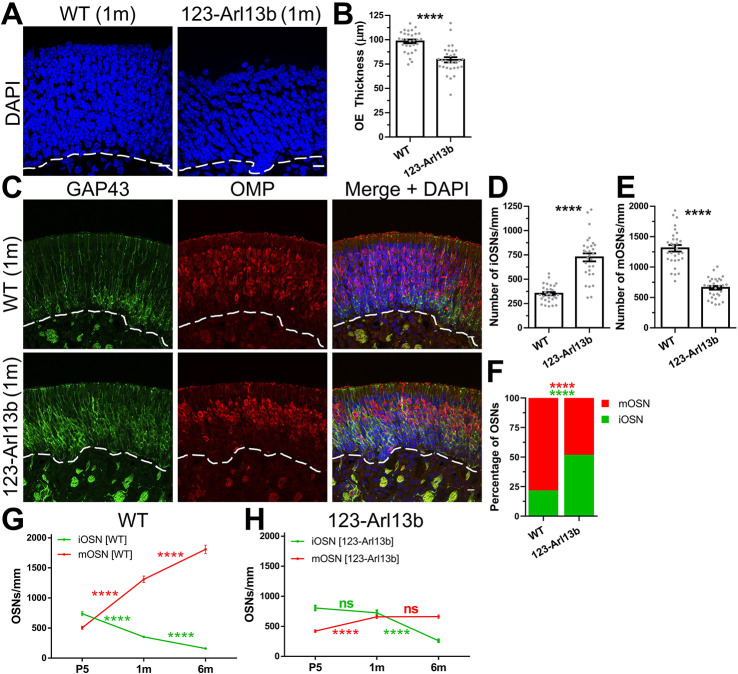
**Loss of ARL13B in iOSNs leads to improper OE development.** (A) Coronal sections of the nasal cavity, stained for DAPI in 1-month-old mice. (B) Quantification of the thickness of the OE (WT, 79.38±2.61 μm; *123-Arl13b*: 98.32±1.93 μm). (C) Immunofluorescence staining of the OE of 1-month-old WT and *123-Arl13b* mice, with GAP43 and OMP labeling. (D) Quantification of the number of iOSNs per mm (WT, 352±16.05 iOSNs/mm; *123-Arl13b*, 725.0±40.99 iOSNs/mm). (E) Quantification of the number of mOSNs per mm (WT, 1310±53.88 mOSNs/mm; *123-Arl13b*, 659.3±30.50 mOSNs/mm). (F) Quantification of the ratio of iOSNs to mOSNs (WT, 21.71/78.29±1.10; *123-Arl13b*, 51.84/48.16±1.42). (G,H) Line graphs showing the number of iOSNs in green and mOSNs in red in WT (G) and *123-Arl13b* (H) mice at P5 and 1 and 6 months of age. Dashed lines delineate basement membrane. Scale bars: 10 μm. Images are representative of ten samples per mouse analyzed. *n*=3 mice for every group. ****P<0.0001 (unpaired, two-tailed Student's *t*-tests). ns, not significant (*P*>0.05). Values represent mean±s.e.m.

We explored the consequence of the loss of ARL13B in iOSNs on the integrity of the OE. DAPI (4′,6-diamidino-2-phenylindole) labeling of coronal sections of 1-month-old mice showed that *123-Arl13b* mice had reduced thickness of the OE compared with WT (Fig. 3A,B). Given this finding, we hypothesized that the thinner OE in *123-Arl13b* mice was due to a loss of iOSNs. To test this hypothesis, the number of OSNs was assessed by immunostaining for GAP43 and OMP. Surprisingly, there was an increase in the number of iOSNs and a decrease in the number of mOSNs in *123-Arl13b* mice compared with WT mice (Fig. 3C-E). On average, the neuronal composition of 1-month-old WT mice consisted of 25% iOSNs and 75% mOSNs whereas in *123-Arl13b* mice the percentages of iOSNs and mOSNs were roughly equal (Fig. 3F). Finally, when comparing 1-month-old WT (*123-Cre^+/+^;Arl13b^fl/fl^*) and *123-Cre* (*123-Cre^+/−^*) mice there was no difference in either the number of iOSNs or mOSNs (Fig. S2), suggesting that the change in OE neuronal composition was not a cytotoxic effect from Cre recombinase expression. These data indicate that the excision of *Arl13b* in iOSNs in 1-month-old mice caused a shift in the neuronal population, with increased iOSNs and a loss of mOSNs.

Uniquely, the OE is a neuroepithelium that matures postnatally and reaches a homeostatic cellular composition in mice at approximately 2 months of age (Weiler and Farbman, 1997). To understand the role of neuronal primary cilia in the development of the OE, the OSN composition of the OE in *123-Arl13b* mice at P5 and 1 and 6 months of age was compared (Fig. 3G,H; Fig. S3). In WT mice, the maturation of the OE was reflected in the continuous increase in number of mOSNs and the reciprocal decrease in the number of iOSNs from P5 to 6 months of age (Fig. 3G). However, the dynamics were dramatically shifted in *123-Arl13b* mice, in which there was a sustained, elevated level of iOSNs from P5 to 1 month of age (no statistical difference) and then a decline at 6 months of age (Fig. 3H). The inverse relationship was seen with *123-Arl13b* mOSNs; there was an initial increase in the number of mOSNs from P5 to 1 month of age, and a plateau from 1 to 6 months of age, with no change in mOSNs (Fig. 3H). Of note, there was no statistical difference in the number of iOSNs at P5 in WT compared with *123-Arl13b* mice (Fig. S3B), suggesting that the initial formation of iOSN is unaffected and then subsequently followed by an over-population of iOSNs **(**1 month old, Fig. 3D; 6 months old, Fig. S3F). A final noteworthy point is that in *123-Arl13b* mice the number of iOSNs decreased whereas the number of mOSNs remained the same from 1 to 6 months of age (Fig. 3H); the lack of compensation suggests a loss in neurons in *123-Arl13b* mice. Together, these results indicate that the OE in *123-Arl13b* mice was immaturely developed at a time when the OE would typically attain homeostasis.

### *Arl13b* mutant OSNs have a delay in neuronal maturation

**Fig. 4.**
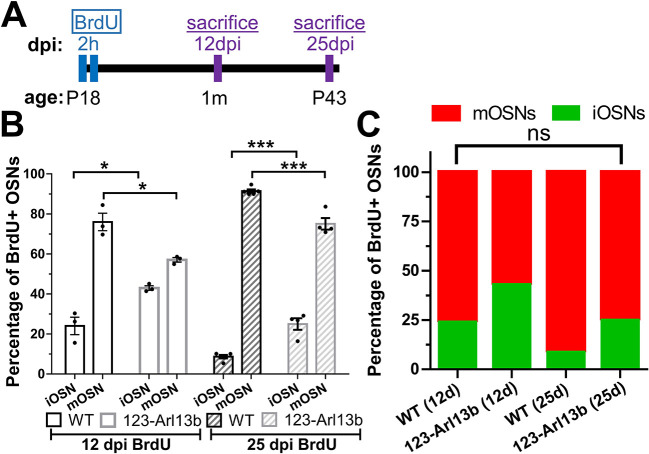
***123-Arl13b* mice have a delay in OSN maturation.** (A) Schematic of BrdU birthdating paradigm. At P18, mice received two i.p. injections of BrdU that were 2 h apart. Mice were perfused at 12 dpi (1 month of age) and 25 dpi (P43). (B) Graph of the percentage of BrdU^+^ OSNs that are either iOSNs or mOSNs in WT and *123-Arl13b* mice at 12 dpi of BrdU (12 dpi: WT, 24.01±4.384% iOSN, 75.99±4.385% mOSN; 12 dpi: *123-Arl13b*, 42.95±1.112% iOSN; 57.05±1.112% mOSN) and at 25 dpi of BrdU (25 dpi: WT, 8.640±2.722% iOSN; 91.39±2.722% mOSN; 25 dpi: *123-Arl13b*, 24.93±2.722% iOSN; 75.07±2.722% mOSN). (C) Stacked graph of the percentage of BrdU-positive OSNs. *n*=3 for both genotypes at 12 dpi and *n*=5 WT, *n*=4 *123-Arl13b* mice at 25 dpi. *P<0.01, ***P<0.001 (unpaired, two-tailed Student's *t*-tests). ns, not significant (*P*>0.05). Values represent mean±s.e.m.

Given the profound defects in the development of the OE in *123-Arl13b* mice, we hypothesized that OSN maturation may be impaired or even halted. We performed a 5-bromo-2′deoxyuridine (BrdU) birthdating experiment, where we administered the synthetic nucleotide BrdU, which incorporates into actively dividing cells in the DNA synthesis (S) phase (Lengronne et al., 2001) by intraperitoneal (i.p.) injection. BrdU was injected in P18 mice two times separated by a 2-hour period. The mice were euthanized either 12 days post-injection (dpi) at 1 month of age or 25 dpi at P43 (Fig. 4A). The time frame was chosen because it takes cells an average of 10-12 days from basal cell division to become mOSNs (Liberia et al., 2019). Following tissue processing, we stained sections of the OE for iOSN and mOSN markers to determine their co-labeling with BrdU (Fig. S4). At 12 dpi, there was a higher percentage of BrdU-positive iOSNs (BrdU^+^/GAP43^+^) and a lower percentage of BrdU-positive mOSNs (BrdU^+^/OMP^+^) in *123-Arl13b* mice compared with WT mice (Fig. 4B). A greater number of BrdU-positive OSNs progressed to a mature state at the 25 dpi compared with the 12 dpi time point in both WT and *123-Arl13b* mice, indicating that OSN maturation was not halted. Nevertheless, as in the 12 dpi condition, at 25 dpi there was still a higher percentage of OSNs that were BrdU^+^/GAP43^+^ in *123-Arl13b* mice relative to the WT mice. Interestingly, the percentages of BrdU^+^/GAP43^+^ and BrdU^+^/OMP^+^ neurons in WT at 12 dpi were the same as those in *123-Arl13b* mice at 25 dpi (Fig. 4C), suggesting that *123-Arl13b* neurons take a little over twice as long to mature than WT OSNs. Overall, these experiments show that loss of ARL13B in iOSNs causes a delay in neuronal maturation.

### Loss of ARL13B in iOSNs does not impair HH or Wnt signaling

**Fig. 5.**
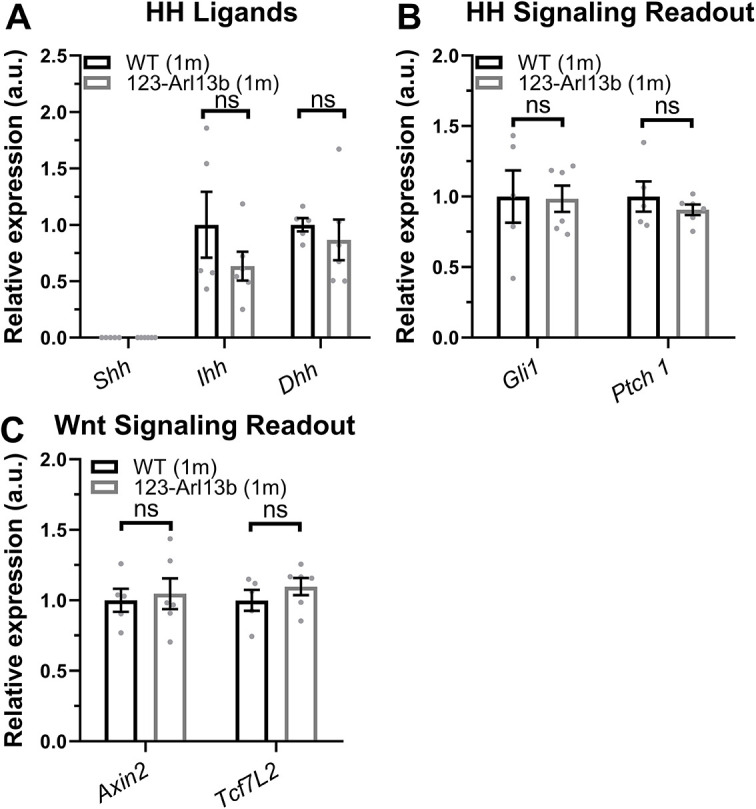
**Loss of *Arl13b* in iOSNs does not change gene expression of HH and Wnt signaling.** (A,B) Quantification of the relative expression of genes encoding HH ligands (*Shh*, *Ihh*, *Dhh*) (A) and HH pathway readouts (*Gli1*, *Ptch1*) (B) in the olfactory mucosa of 1-month-old WT and *123-Arl13b* mice. *Shh* transcript was not detected and therefore the relative expression was set to 0. (C) Relative expression of Wnt signaling readouts (*Axin2*, *Tcf7l2*) in 1-month-old WT and *123-Arl13b* mice. *n*=5 WT, *n*=6 *123-Arl13b* mice. a.u., arbitrary units; ns, not significant (*P*>0.05; unpaired, two-tailed Student's *t*-tests). Values represent mean±s.e.m.

Primary cilia are specialized organelles that are essential sites for the signal transduction of many pathways, including HH and Wnt signaling (Wheway et al., 2018). In various cell types, loss of ARL13B results in low level constitutive activation of HH (Caspary et al., 2007; Larkins et al., 2011; Ferent et al., 2019; Shao et al., 2017). Therefore, we tested gene expression of the HH ligands sonic HH (*Shh*), Indian HH (*Ihh*) and desert HH (*Dhh*) and readouts of the pathway (*Gli1*, *Ptch1*) in the olfactory mucosa of *123-Arl13b* mice. *Shh* transcript was not detected [cycle threshold (CT)>38] at P5 and 1 month of age in either genotype (Fig. 5A, Fig. S5A-C), which corroborates earlier findings in P2 WT mice (Gong et al., 2009). We confirm that the previously published (Kopinke et al., 2017) *Shh* primer used was functional as we detected high levels of *Shh* in an embryonic day 11 control (Fig. S5A,C), as expected (Jiang and Hui, 2008; Krauss et al., 1993). Interestingly, we observed detectable levels of *Ihh* and *Dhh* in both P5 and 1-month-old mice, but no differences between WT and *123-Arl13b* mice (Fig. S5A, Fig. 5A). Additionally, at 1 month of age we determined that there was no difference between genotypes in the gene expression of the transcriptional targets of HH, *Ptch1* and *Gli* (Fig. 5B). We repeated the assay in P5 mice and found that the number of iOSNs is the same between WT and *123-Arl13b* mice (Fig. S3B) and corroborated the lack of change in HH signaling gene expression (Fig. S5B). Interestingly, we found that expression of *Ihh*, *Ptch1* and *Gli1* dramatically decreased in WT mice from P5 to 1 month of age (Fig. S5A). We next tested whether there were any changes in Wnt signaling as it has been suggested to play a role in OSN maturation (Wang et al., 2011). However, at either P5 or 1 month of age there was no difference in gene expression of the Wnt pathway readouts *Axin2* and *Tcfl2* (also known as *Tcf4*) between WT and *123-Arl13b* mice (Fig. S5D, Fig. 5C). Given the lack of difference in HH and Wnt gene expression between genotypes, these data suggest that the phenotypes observed in the *123-Arl13b* murine model are generated through a HH- and Wnt-independent mechanism.

### Loss of *Arl13b* in iOSNs increased cell death and proliferation in the OE

**Fig. 6.**
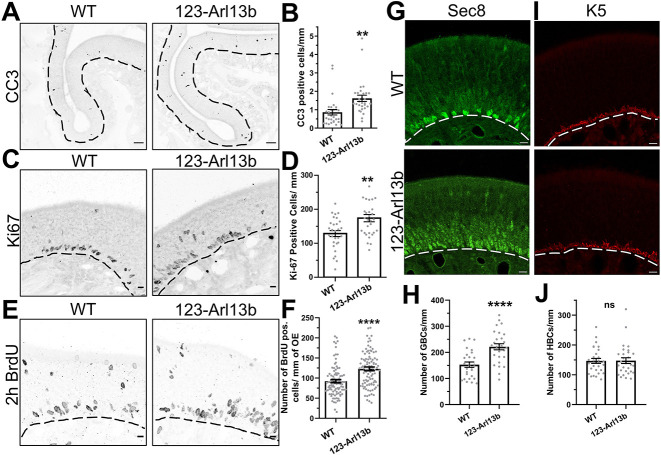
***123-Arl13b* mice exhibit increased cell death and proliferation in the OE.** (A) Representative images of the CC3-immunolabeled OE of 1-month-old mice. (B) Quantification of the number of CC3^+^ cells/mm (WT, 0.865±0.147 CC3^+^ cells/mm; *123-Arl13b*, 1.620±0.170 CC3^+^ cells/mm). (C) Representative images of Ki67 immunolabeling in the OE of 1-month-old mice. (D) Quantification of Ki67^+^ cells per mm (WT, 128.7±8.719 Ki67^+^ cells/mm; *123-Arl13b*, 174.1±10.58 Ki67^+^ cells/mm). (E) Representative images of the OE labeled for BrdU, 2 h after i.p. injections of BrdU at P18. (F) Quantification of the number of BrdU^+^ cells (WT, 92.20±3.897 BrdU^+^ cells/mm; *123-Arl13b*, 122.9±4.738 BrdU^+^ cells/mm). (G,I) Staining of the stem cells of the OE, by Sec8 (G) and K5 (I) immunohistochemistry in 1-month-old mice. (H) Quantification of the number of GBCs per mm (WT, 152.8±10.17 cells/mm; *123-Arl13b*, 221.4±11.66 cells/mm). (J) Quantification of the number of HBCs per mm (WT, 146.4±8.371 HBCs/mm; *123-Arl13b*, 147.5±9.669 HBCs/mm). Dashed lines delineate basement membrane. Scale bars: 10 μm (C,E,G,I); 20 μm (A). Images are representative of ten samples per mouse analyzed. *n*=3 mice for both groups (B,D,H,J), *n*=5 for both genotypes (F). **P<0.01, ****P<0.0001 (unpaired, two-tailed Student's *t*-tests). ns, not significant (*P*>0.05). Values represent mean±s.e.m.

We next investigated whether the delay in the maturation of OSNs observed in the *123-Arl13b* mice caused a dysregulation in the homeostatic balance between OE cell loss and proliferation. We explored the effects of cell death with the apoptotic marker cleaved-caspase three (CC3). There was an increase in CC3-expressing cells in *123-Arl13b* mice compared with WT at 1 month of age (Fig. 6A,B). Subsequently, proliferation was assayed by using two well-established markers of cell division, endogenous Ki67 (Mki67) (Scholzen and Gerdes, 2000) and incorporated BrdU (Gratzner, 1982). Ki67 immunolabeling was performed in sections of the nasal cavity of 1-month-old mice. For the BrdU proliferation assay, P18 mice received two i.p. injections 2 h apart and were then euthanized 2 h later. Both assays showed that proliferation increased in the OE of *123-Arl13b* mice compared with WT mice (Fig. 6C-F). Furthermore, the OE stem cell populations in *123-Arl13b* mice were also evaluated. Tissue sections were stained for HBCs and GBCs using the markers K5 and Sec8 (Exoc4), respectively. Compared with WT mice, *123-Arl13b* mice had a statistically significant increase in the number of GBCs, the mitotically active stem cells (Fig. 6G,H). As expected, there was no difference between both genotypes in the number of HBCs, the homeostatically quiescent stem cells (Fig. 6I,J). Together, the data show that loss of *Arl13b* in iOSNs leads to an increase in cell death and proliferation in the OE.

Given the dysregulation of various processes, including cellular maturation, proliferation and death, we investigated whether the delay in neuronal maturation was the precipitating event. We utilized the olfactotoxin methimazole (MMZ) to ablate and thus reset the OE by i.p. injection in 1-month-old mice. Additionally, to birthdate regenerating cells mice were injected intraperitoneally with BrdU twice, 1 day and 2 days after MMZ administration. Mice were euthanized 1 month after MMZ administration. Interestingly, when assaying the cellular composition of the OE the only defect observed was a decrease in mOSNs in *123-Arl13b* mice compared with WT (Fig. S6A,C). We detected no change in the number of iOSNs (Fig. S6A,B), GBCs (Fig. S6D,E) and HBCs (Fig. S6F,G) between genotypes. There also was no change in cell death in the regenerating OE between WT and *123-Arl13b* mice (Fig. S6H,I). However, the BrdU birthdating experiments revealed an increase in the percentage of BrdU^+^/GAP43^+^ OSNs and a decrease in BrdU^+^/OMP^+^ OSNs in *123-Arl13b* mice compared with WT, showing a delay in OSN maturation (Fig. S6J). These data suggest that the loss of ARL13B in iOSNs first leads to a delay in maturation with a resulting decrease in mOSNs.

### Odor detection was impaired after loss of *Arl13b* in iOSNs

**Fig. 7.**
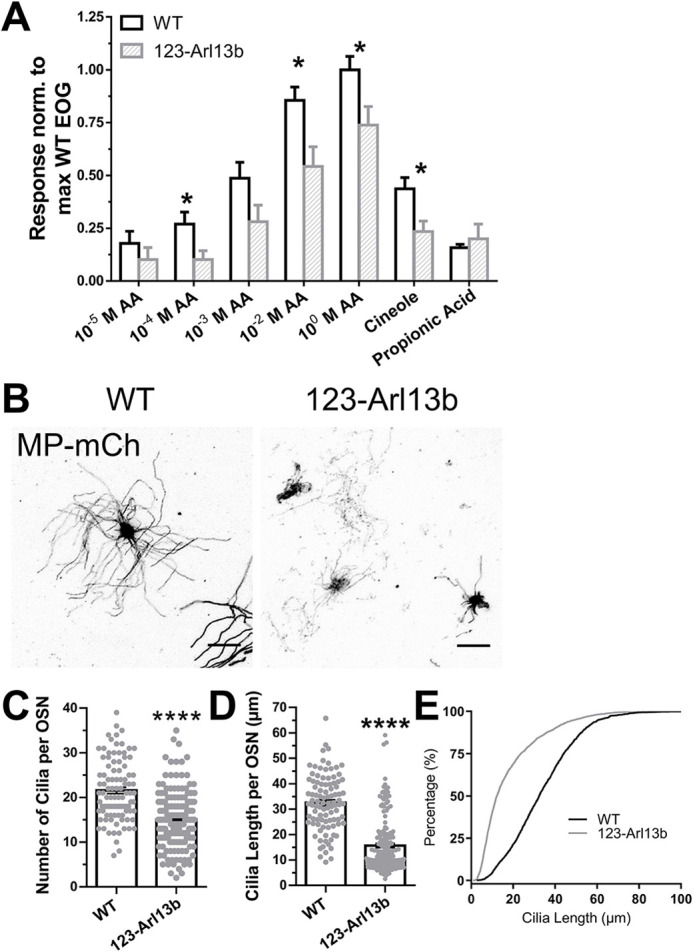
***123-Arl13b* mice have impaired odor detection and defective OSN ciliation.** (A) EOG recordings of a dose escalation of AA and other odorants (cineole and propionic acid) at the same vapor pressure (*P_vapor_*) as 10^−3^ M AA, normalized to the average WT 10^0^ M AA response. (B) Representative *en face* confocal images of mOSN cilia marked by AV-MP-mCh of WT (left) and *123-Arl13b* mice (right). (C) Quantification of the average number of cilia per OSN (WT, 21.58±0.6885 cilia; *123-Arl13b*: 15.05±0.4860 cilia). (D) Quantification of the average length of cilia per OSN (WT, 32.51±1.112 μm; *123-Arl13b*, 15.69±0.851 μm). (E) Quantification of the distribution of cilia length per OSN. EOG studies (A): *n*=9 mice for both groups; ciliation experiments (B-E): WT: *n*=97 OSNs in 7 mice; *123-Arl13b*: *n*=164 OSNs in 6 mice. Scale bars: 10 μm. Images are representative of ten samples per mouse analyzed. Two-month-old mice were used for all experiments. *P<0.05, ****P<0.0001 (unpaired, two-tailed Student's *t*-tests). Values represent mean±s.e.m.

Because of the profound defects in the OE, we assayed odor detection in *123-Arl13b* mice using electro-olfactogram (EOG) recordings. A concentration escalation of the odorant amyl acetate (AA) showed impaired odor detection at the higher concentrations (Fig. 7A). Testing of single odorants at the same vapor pressure as 10^−3^ M AA showed that delivery of cineole led to a statistically significant decrease in EOG response compared with WT, which was not observed for propionic acid delivery (Fig. 7A). Given the delay in maturation, at a time when OSN multi-ciliation occurs and the hyposmic phenotype of the*123-Arl13b* mice, we next tested whether the loss of ARL13B in iOSNs impacts ciliation of mOSNs. Representative *en face* images showed that *123-Arl13b* mOSNs had fewer and shorter cilia compared with WT OSNs, which was supported by quantification (Fig. 7B-E). Therefore, the loss of ARL13B in iOSNs resulted in diminished odor detection ability and reduced ciliation of mOSNs.

### Loss of *Arl13b* in iOSNs causes a deformation in glomerular shape and diminished OSN innervation of glomeruli

**Fig. 8.**
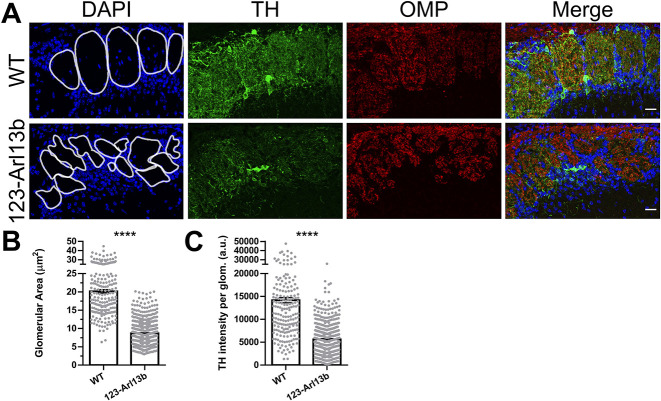
**Loss of ARL13B in iOSNs leads to improper glomerular innervation.** (A) Coronal sections of the OB of 1-month-old WT and *123-Arl13b* mice stained for DAPI, TH and OMP. (B) Quantification of the glomerular area (WT, 20.15±0.4608 μm^2^; *123-Arl13b*, 8.676±0.1470 μm^2^). (C) Quantification of the TH intensity after subtracting the background intensity (tissue autofluorescence) (WT, 14,180±563.0 a.u.; *123-Arl13b*, 5628±153.5 a.u.). White lines outline the glomerular perimeter (A, left). a.u., arbitrary units. *n*=2 WT mice; *n*=3 *123-Arl13b* mice. Scale bars: 20 μm. Images are representative of ten samples per mouse analyzed. ****P<0.0001 (unpaired, two-tailed Student's *t*-tests). Values represent mean±s.e.m.

OSNs are bipolar neurons with cilia, the site of odor detection, residing peripherally in the OE and axons that extend and synapse in glomeruli in the OB. Given the defects in odor detection in *123-Arl13b* mice, we investigated whether the delay in maturation impacted the innervation of glomeruli. In 1-month-old mice, coronal sections of the OB were stained for: DAPI to delineate glomeruli; OMP to visualize axonal convergence in glomeruli; and tyrosine hydroxylase (TH), a surrogate marker of afferent neuronal activity. *123-Arl13b* glomeruli were smaller than those of WT, and intriguingly, were severely deformed, as evidenced both by DAPI and OMP staining (Fig. 8A,B). Also, there was a reduction in TH intensity in the glomeruli of *123-Arl13b* compared with WT mice (Fig. 8A,C). The axons of OSNs fasciculate as they traverse from the periphery and form the outermost layer of the OB, known as the olfactory nerve layer (ONL). We stained coronal sections of the OB for OMP. We measured a statistically significant decrease in ONL thickness and area in 1-month-old *123-Arl13b* mice compared with WT (Fig. S7), which may be reflective of the loss of mOSNs. Together, these data indicate that there is a functional consequence to the loss of ARL13B in iOSNs that manifests in the OB, as a deformation of glomeruli and impaired glomerular innervation in 1-month-old mice.

**Fig. 9.**
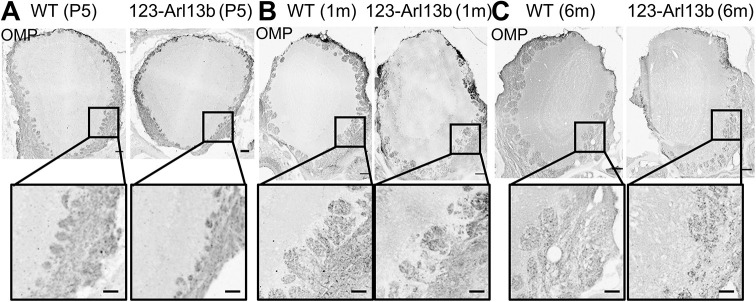
**Developmentally regulated deformation of glomerular structure in *123-Arl13b* mice.** (A-C) Representative whole-stitch images of coronal sections of OBs immunolabeled with OMP in P5 (A), 1-month-old (B) and 6-month-old (C) WT and *123-Arl13b* mice. Insets show higher magnification images of glomeruli. Scale bars: 50 μm (insets); 100 μm (main panels). Images are representative of ten samples per mouse analyzed.

The OB develops postnatally through a process that involves the formation and enlargement of glomeruli (Pomeroy et al., 1990). Whole-stitch images of the OB, immunolabeled for OMP were collected to investigate changes in the innervation of glomeruli at P5 and 1 and 6 months of age in *123-Arl13b* mice. At all ages, WT glomeruli were clearly visible as distinct structures throughout the outer perimeter of the OB; from P5 (Fig. 9A, left) to 6 months of age (Fig. 9C, left), the size and number of glomeruli increased. At P5, the glomeruli in WT and *123-Arl13b* mice were similar in shape and distribution along the OB; however, the glomeruli in P5 *123-Arl13b* mice appeared smaller compared with WT (Fig. 9A). In 1- and 6-month-old *123-Arl13b* mice, the arrangement of the glomeruli was disorganized and there were multiple regions with missing glomeruli (Fig. 9B,C). These data indicate that in *123-Arl13b* mice glomeruli form normally and subsequently degrade by 1 month of age.

## DISCUSSION

We show for the first time that ARL13B has a maturation state-dependent expression in OSNs, where it is expressed in primary cilia in iOSNs, but excluded from the multi-cilia of mOSNs. Utilizing a novel murine model, an iOSN-specific knockout of *Arl13b* (*123-Arl13b*), we demonstrate that ARL13B is necessary for the proper development of the OE. The loss of *Arl13b* in iOSNs resulted in a profound delay in neuronal maturation. This postponement manifested in shorter and fewer cilia on mOSNs, diminished odor detection, and glomeruli with structural deformations. Overall, these findings highlight the importance of the regulatory ciliary GTPase ARL13B on the maturation of OSNs and the development of the OE.

Several reports have identified that OSNs possess an initial, short cilium that has unique characteristics distinct from those of the subsequent cilia on OSNs. The first cilium on OSNs was determined to possess an axoneme lacking the central pair of singlet microtubules that is characteristic of primary cilia (Menco and Farbman, 1985a,b), to be devoid of odorant receptor expression (Schwarzenbacher et al., 2005), and to have a basal body with pre-existing appendages that presumably was a parental centriole (Ching et al., 2022). This study confirms the presence of the primary cilium on neurons in the OE through the use of the canonical primary cilia marker ARL13B, specifically with both the *Arl13b-GFP^tg^* reporter mouse and staining for endogenous ARL13B. A seminal, yet surprising, discovery in this study was the expression of ARL13B in the primary cilia of iOSNs, but not in the multi-cilia of mOSNs. Importantly, proteomic databases of isolated cilia of mOSNs did not identify ARL13B among the ciliary proteins (Mayer et al., 2009; Kuhlmann et al., 2014; Stephan et al., 2009; McClintock et al., 2008). The finding was remarkable given the presence of ARL13B in the multi-ciliated cells of the RE and other multi-ciliated cells, such as cells in ependyma, upper airway, and lung (Ha et al., 2016; Cevik et al., 2013). Intriguingly, the loss of ARL13B in iOSNs resulted in mOSNs with shorter and fewer cilia compared with WT mOSNs. Our data support the documented role of ARL13B on cilia length (Caspary et al., 2007; Higginbotham et al., 2012; Thorpe et al., 2017) and ciliation (Hori et al., 2008; Larkins et al., 2011). Given that ARL13B is not present in the cilia of mOSN, we hypothesize that ARL13B in OSNs exerts control over cilia biogenesis. The unique expression of ARL13B in the primary cilium of iOSNs makes it a powerful and useful tool to not only identify but also manipulate the primary cilium. A theory emerges whereby ARL13B-mediated signaling through the primary cilium may play a role in the progression of OSNs from a single ciliated to a multi-ciliated cell.

In the olfactory system, a concerted effort has been placed on determining molecular cues and markers that are integral for the differentiation of the multipotent progenitor cells in the OE (Schwob et al., 2017); however, the signaling mechanisms necessary for the maturation of OSNs have been underexplored. What is known about the post-mitotic maturation of OSNs (McClintock et al., 2020) is the following: (1) the crucial biological processes within the major stages of OSN differentiation and the timing of both (Fletcher et al., 2017; Hanchate et al., 2015; Nickell et al., 2012; Heron et al., 2013; Saraiva et al., 2015); (2) molecular markers; and (3) the morphological changes during neuronal maturation (Liberia et al., 2019; Rodriguez-Gil et al., 2015; Savya et al., 2019). Given that the expression of ARL13B is dependent on the maturation state of OSNs, we hypothesized that ARL13B in the primary cilium might play a role in neuronal maturation. Using a series of BrdU birthdating assays, we demonstrated that loss of ARL13B in iOSNs led to a delay in maturation. Another protein highly abundant in cilia, adenylate cyclase 3 (AC3; ADCY3), which is expressed during OSN maturation, has been suggested to play a role in OSN maturation (Zhang et al., 2017). We note various phenotypic similarities between AC3^−/−^ and *123-Arl13b* mice, including OE cellular composition (increase in iOSNs and decrease in mOSNs) and increased cell death. Odor detection in *123-Arl13b* mice was reduced but not abolished like in the AC3^−/−^ mice. This was presumably due to loss of overall OSN ciliation in *123-Arl13b* mice whereas in the AC3^−/−^ mice a crucial component of the olfactory signaling transduction pathway was knocked out. Regardless, both murine models resulted in loss of sensory input, albeit more severely in AC3^−/−^ mice. It may therefore be hypothesized that the impact of both ARL13B and AC3 on maturation was a result of sensory deprivation. Additionally, the crucial process of synaptogenesis occurs during OSN maturation and recently the exuberant and plastic nature of the iOSN synapse has been revealed (Cheetham et al., 2016; Huang et al., 2022; Marcucci et al., 2011). Perhaps both peripheral odorant detection and the subsequent synaptic transmission are necessary for the maturation of an OSN.

Importantly, the predominant function of the primary cilium is to act as ‘signaling antenna’ (Berbari et al., 2009) and as such it controls numerous pathways, including Wnt and HH signaling (Wheway et al., 2018). Clues from other neuronal tissues and organs (Rosso and Inestrosa, 2013) prompted the study of Wnt signaling in OSN neurogenesis. Studies showed that Wnt signaling in the OE is important for the proliferation and differentiation of stem cells into neuronally fated cells as well as playing a key role in OSN axon outgrowth (Wang et al., 2011; Rodriguez-Gil and Greer, 2008). A study qualitatively stated that within a subpopulation of GBCs, those that express Lgr5, the impairment of Wnt signaling caused a delay in OSN maturation (Chen et al., 2014). Also, the mutation of ARL13B caused a loss of WNT ligand in the neural tube (Horner and Caspary, 2011; Caspary et al., 2007). However, we observed no changes in gene expression of Wnt signaling in the *123-Arl13b* mouse model, suggesting that the maturation of OSNs regulated by ARL13B is not through the Wnt signaling pathway. Given that Wnt signaling is predominantly activated in HBCs and GBCs but infrequently in iOSNs (Wang et al., 2011), Wnt signaling in the OE likely plays a role in stem cell differentiation rather than maturation. Overall, the newly derived, olfactory system-specific *Arl13b* null mouse model may provide a useful tool to ascertain the genetic programs that regulate the maturation of OSNs.

There is a well-documented relationship between ARL13B and the cilia-specific pathway, HH signaling (Caspary et al., 2007; Gigante et al., 2020; Larkins et al., 2011; Mariani et al., 2016; Su et al., 2012). Therefore, it was reasonable to hypothesize that ARL13B in iOSNs controls neuronal maturation through a HH-dependent pathway. Additional support for this hypothesis was provided in a study conducted in the olfactory system, which noted that loss of the HH ligand SHH in a SHH knockout mouse model led to an apparent delay in the rate of OSN maturation (Balmer and LaMantia, 2004). Interestingly, even though loss of *Arl13b* in various cell types resulted in a decrease of *Gli* transcriptional activation (Caspary et al., 2007; Su et al., 2012; Bay et al., 2018), we detected no difference in gene expression of either the HH ligands or HH activity between WT and *123-Arl13b* mice. These findings suggest that the HH signaling pathway is not involved in the ARL13B-mediated maturation of OSNs. We cannot definitively exclude the possibility of an ARL13B-HH mechanism during development given that the role of HH in the olfactory system has been reported during embryogenesis (Balmer and LaMantia, 2004; Gong et al., 2009; Persson et al., 2014), a time at which we did not assay HH activity. However, given that the *123-Arl13b* phenotype exacerbates with age and that HH activity dramatically declines by 1 month of age we find this possibility unlikely. We surmise that the phenotypes observed after loss of *Arl13b* in iOSNs are likely not generated through the canonical HH pathway, which is divergent from the numerous reports of the regulation of HH signaling by ARL13B.

There is mounting evidence relating to the importance of the primary cilium in the development of the mammalian nervous system through direct and modulatory control of signaling pathways crucial for proliferation and neurogenesis (Guemez-Gamboa et al., 2014). Loss of the primary cilia protein ARL13B causes defects in the maturation of neuronal tissues and neuronal patterning (Higginbotham et al., 2012; Caspary et al., 2007). In this study, the modulation of the primary cilium through loss of ARL13B in iOSNs resulted in an increase in neurogenesis, proliferation and number of GBCs. Given that GBCs were not directly impacted in the *123-Arl13b* mouse model, the increase in GBCs and proliferation was presumably through an indirect mechanism. Additionally, *123-Arl13b* mice never achieved the WT homeostatic cellular composition of the OE and thus never properly developed. Through the use of an olfactotoxin, the OE was ablated and it was determined that the loss of ARL13B first led to the delay in maturation with a resulting decrease in mOSNs. This would suggest that the loss of cells, increase in proliferation, and increase in GBCs resulted from the initial delay in maturation. The consortium of defects in the OE of *123-Arl13b* mice all reveal an impairment in the homeostatic balance of neurogenesis and cell death. There is evidence to support a negative feedback loop whereby GBCs in the OE are able to sense the number of OSNs and adjust their rate of proliferation and differentiation accordingly (Mumm et al., 1996; Calof et al., 1996). Presumably, the lack of mOSNs caused the GBCs to then proliferate, increasing the iOSN pool in *123-Arl13b* mice; however, it remains unclear whether the inhibition of stem cell proliferation and differentiation by OSNs is dependent on the maturation state of the OSN. Perhaps other inhibitory processes are responsible for the homeostatic imbalance in *123-Arl13b* mice. Studies have suggested several factors that may control neurogenesis and stem cell differentiation, including FGF8, GDF11 and FST (Kawauchi et al., 2005; Wu et al., 2003; DeHamer et al., 1994); it will be important to determine whether the primary cilium on iOSNs influences these various factors.

It is well documented that ciliopathy mouse models display glomerular innervation defects, with smaller glomeruli and a decrease in afferent activity (Green et al., 2018; Uytingco et al., 2019b; Williams et al., 2017; Xie et al., 2021; Tadenev et al., 2011). We found that loss of the JS gene *Arl13b* in iOSNs also resulted in smaller glomeruli and reduced TH expression, which is widely correlated with OSN activation in the OB (Baker et al., 1984). Given that *123-Arl13b* mice suffer from impaired odor detection and diminished ciliation, the glomerular defects may be explained by the dynamic reciprocity between sensory input and glomerular stability (Schwob et al., 1992, 1993, 1994, 2001). Remarkably, *123-Arl13b* mice displayed glomeruli with anatomical deformations that were not observed in the glomeruli of other ciliopathy mouse models (Green et al., 2018; Uytingco et al., 2019b; Williams et al., 2017; Xie et al., 2021; Tadenev et al., 2011). The irregularity in OSN axon glomerular convergence is reminiscent of findings in which OSNs were killed by genetic expression of diphtheria toxin (Child et al., 2018) or axotomy (Murai et al., 2016). The increased cell death and loss of mOSNs in *123-Arl13b* mice may be responsible for the malformation of glomeruli. Several studies in the olfactory system have reported that HH signaling exerts control over axon extension and pathfinding as well as in the development and stability of glomeruli (Balmer and LaMantia, 2004; Gong et al., 2009; Persson et al., 2014). However, we observed no changes in HH activation in the olfactory mucosa where OSN soma reside. Furthermore, the aforementioned studies discovered a developmental role of HH in glomeruli, and in the present study the glomeruli appear to form properly although they are smaller, presumably owing to the decrease in mOSNs, and then become deformed with age. These findings pose a perplexing question of how a protein localized to a primary cilium in the periphery causes axonal defects in the central nervous system.

Over 35 genetic disorders in humans affect the structure, function, and/or maintenance of cilia, collectively termed ciliopathies (Waters and Beales, 2011; Reiter and Leroux, 2017). We and others have shown that ciliopathies result in olfactory dysfunction in humans and in animal models (Green et al., 2018; McIntyre et al., 2012; Uytingco et al., 2019a,b; Williams et al., 2017; Tadenev et al., 2011; Xie et al., 2021). Individuals suffering from the ciliopathy JS experience a myriad of symptoms, including polycystic kidney disease, retinal degeneration, deficiencies in cognitive function, and the hallmark molar tooth sign caused by cerebral malformations (Cantagrel et al., 2008). It is unclear whether JS patients suffer from olfactory dysfunction because deficits in their cognitive capacity impede proper clinical diagnosis of smell impairment, which requires psychophysical evaluation (Brancati et al., 2010; Whitcroft and Hummel, 2019). One of the more severe forms of JS is caused by mutation of *ARL13B* (Caspary et al., 2007; Cantagrel et al., 2008). Importantly, the olfactory deficits induced by loss of ARL13B in iOSNs have potentially profound implications for our understanding of olfactory function in individuals with JS.

## MATERIALS AND METHODS

### Mouse strains and genotyping

The mice in this study were housed in a standard animal facility at the University of Florida. All procedures were approved by the University Committee for the Use and Care of Animals. A transgenic reporter mouse with *Arl13b* tagged with GFP, referred to as *Arl13b-GFP^tg^* (Delling et al., 2013), was generously provided by Dr David Clapham (Harvard University, Cambridge, MA, USA). *123-Cre* mice (*123-Cre*, also known as *Goofy-Cre*) (Hirata et al., 2006) were kindly provided by Dr Yoshihiro Yoshihara (RIKEN Brain Science Institute, Japan) and the *Arl13b* floxed mice (*Arl13b^fl/fl^*) (Su et al., 2012) were generously gifted by Dr Tamara Caspary (Emory University, Atlanta, GA, USA). The *123-Arl13b* mouse model was generated by crossing a *Arl13b^fl/fl^* mouse with a *123-Cre* mouse that expresses Cre recombinase specifically under the *123* promoter in immature OSNs. Homozygous *Arl13b* floxed mice carrying a *123-Cre* allele were used as *123-Arl13b* mutants (*123-Cre^+/−^;Arl13b*^Δ/Δ^). Littermate mice that did not possess any *Cre* allele (*123-Cre^+/+^;Arl13b^fl/fl^*) were used as controls in all experiments. The genotyping primers for *Arl13b^fl/fl^* were as follows (from 5′ to 3′): Arl13b-LoxP forward ACTCTGGCTTCTTGGTGTCC; Arl13b-loxP reverse CCAGCTTGGGTTATTTCCTGT. For *123-Cre* genotyping, the primers were (from 5′ to 3′): WT forward TTACGTCCATCGTGGACAGC; WT reverse TGGGCTGGGTGTTAGCCTTA; mutant forward GAACCTGATGGACATGTTCAGG; mutant forward AGTGCGTTCGAACGCTAGAGCCTGT. All mice had a mixed genetic background. WT littermates of mutant mice were used as controls. Primers and PCR parameters used in this study for genotyping were previously published and are referenced above.

### Tissue collection and preparation

Mice were anesthetized with xylazine (100 mg/kg) and ketamine (10 mg/kg), transcardially perfused with 4% paraformaldehyde and decapitated. Dissected snouts were post-fixed in 4% paraformaldehyde for 12-16 h at 4°C. The tissue was then decalcified in 0.5 M EDTA in 1×PBS for 3-5 days (depending on age) at 4°C on a shaker and cryoprotected in sequential solutions of sucrose at 10% (2 h), 20% (2 h) and 30% (overnight) in a 1×PBS solution at 4°C on a shaker. Next, the tissue was frozen in OCT compound (Tissue-Tek) and allowed to solidify in a −80°C deep freezer for 12-16 h. Coronal sections of the nasal cavity and OB were cut using a CM1860 Leica cryostat (Leica Biosystems) at 10-12 μm thickness onto microscope slides and stored in a −20°C freezer.

### Immunofluorescence staining

For immunofluorescence staining, tissue sections were allowed to thaw for 15 min at room temperature (T_rm_) and then rinsed three times for 5 min each wash in 1×PBS to remove OCT and rehydrate sections. If staining required antigen retrieval (AR), slides were incubated in citric acid buffer, pH 6.0, for 30 min at 95°C, cooled for 20 min at T_rm_, and washed with distilled water for 5 min. The following primary antibodies needed AR: anti-Sec8, anti-GAP43, anti-BrdU and anti-ARL13B. All tissue sections were blocked with 2% donkey serum, 1% bovine serum albumin and 0.1% Triton X-100 in 1×PBS and then incubated overnight with primary antibody in the blocking solution at 4°C. For BrdU staining, an additional step was required after the blocking step in which slides were incubated in 0.02 M HCl for 30 min at 65°C. The following antibodies were used: goat anti-OMP (1:1000, Wako, 544-10001); rabbit anti-TH (1:500, Millipore, AB152); chicken anti-GAP43 (1:1000, EnCor, CPCA-GAP43); mouse ARL13B (1:500, NeuroMab, 75-287), mouse anti-Sec8 (1:200, BD Transduction Laboratories, 610658), rabbit anti-K5 (1:2500, BioLegend, 905501), rat anti-BrdU (1:200, Abcam; ab6326), rabbit anti-CC3 (1:500, Millipore, AB3623) and chicken anti-Ki67 (1:200, EnCor, RPCA-Ki67). Sections were then washed in 1×PBS three times for 5 min each wash at T_rm_ and incubated for 1 h at T_rm_ with Alexa Fluor-conjugated secondary antibodies [donkey anti-mouse Alexa Fluor 488, A-21202 (Invitrogen); donkey anti-mouse Alexa Fluor 594, A-21203 (Invitrogen); donkey anti-goat Alexa Fluor 594, A-11058 (Invitrogen); donkey anti-goat Alexa Fluor 647, A-21447 (Invitrogen); donkey anti-rabbit Alexa Fluor 488, A-21206 (Invitrogen); donkey anti-rabbit Alexa Fluor 594, A-21207 (Invitrogen); donkey anti-chicken Alexa Fluor 488, AB_2340375 (Jackson ImmunoResearch), donkey anti-chicken Alexa Fluor 594, AB_2340377 (Jackson ImmunoResearch), donkey anti-rat Alexa Fluor 594, A-11007 (Invitrogen)] at a concentration of 1:1000. The secondary antibody concentration for staining against Sec8, Ki67 and BrdU was 1:250. After three 5 min rinses in 1×PBS, the tissue sections were incubated with DAPI (1:3000 in 1×PBS) for 10 min, washed three times with 1×PBS, and then sealed with coverslips mounted with ProLong Gold (Invitrogen, P36984).

### Reverse transcription-quantitative polymerase chain reaction (RT-qPCR)

The olfactory mucosa from each naris were dissected, placed in an Eppendorf tube, and drop fixed in liquid nitrogen then stored at −80°C. To isolate RNA, 1 ml of TRIzol reagent (Thermo Fisher Scientific) was added to each sample and the tissue was homogenized using a mortar and pestle. Samples were then vigorously shaken for 15 s and centrifuged at 12,000 ***g*** for 15 min at 4°C. Then 350 μl of the aqueous phase was carefully removed and the RNA was cleaned up using the QIAGEN RNeasy kit. For the embryo control, we purchased total RNA pooled from male and female P11 BALB/c embryos (Takara Bio, 636608). Utilizing the iScript cDNA synthesis kit (Bio-Rad), 500 ng of RNA was used to make cDNA, which was then diluted 100-fold. Using EXPRESS SYBR GreenER 2X master mix (Invitrogen) and 0.4 μl of cDNA in a 384-well plate, quantitative PCR was performed (ABI 7900HT, Applied Biosystems). The experiments were performed in quadruplet. The data were quantified using the comparative CT method (2^−ΔΔCT^ method; Schmittgen and Livak, 2008) and normalized to the housekeeping gene *Pde12*. The primer sequences for qPCR were as follows: *Pde12*, ACCTTTTGGGTGCCAGTAGA, CCAGAGGTCATCTGTCCTTCA; *Shh*, GGCCAAGGCATTTAACTTGT, CCAATTACAACCCCGACATC; *Ihh*, TGACAGAGATGGCCAGTGAG, CAATCCCGACATCATCTTCA; *Dhh,* CGATGGCTAGAGCGTTCAC, GTACCCAACTACAACCCCGA; *Gli1*, GGTGCTGCCTATAGCCAGTGTCCTC, GTGCCAATCCGGTGGAGTCAGACCC; *Ptch1*, AATTCTCGACTCACTCGTCCA, CTCCTCATATTTGGGGCCTT; *Axin2*, AGTGAGACGCTCTCCCTCACCA, GAAACGGGCATAGGTTTGGTGGAC; *Tcf7l2*, CACGCCTCTCATCACGTACA, CCTAGCGGATGGGGGATTTG.

### Live *en face* imaging and OSN cilia measurements

The production of adenovirus-5 MP-GFP and MP-mCherry have been previously described (Williams et al., 2014). Two-month-old mice were anaesthetized with xylazine/ketamine and administered 10 μl of virus on two consecutive days. Following virus delivery, the mice were closely monitored for 30 min and the subsequent days. A detailed procedure of intra-nasal administration to transduce OSNs has been published (Uytingco and Martens, 2019). Ten days after intranasal viral administration, mice were euthanized with CO_2_, decapitated, and the head was split along the cranial midline to expose the OE. The olfactory turbinates were dissected in 1× artificial cerebrospinal fluid, placed in a tissue chamber and held in place with a tissue slice holder. The tissue was placed in a bath of freshly carbogenated (95% O_2_ and 5% CO_2_) artificial cerebrospinal fluid. Confocal *z*-stack images of transduced OSNs were captured using a Nikon A1 confocal microscope with a 60× objective. Quantification of the number of cilia per neuron and length of cilia was carried out using ImageJ/Fiji software (NIH).

### EOG recordings

Mice were euthanized with CO_2_, rapidly decapitated, and the head was hemi-sectioned along the cranial midline. The septal epithelium was removed to expose the olfactory turbinates and the intact hemisected preparation was mounted in a Sylgard-lined chamber. Vapor-phase odor stimuli were generated by diluting 100 μl of odorant (in DMSO) into 10 ml water (final volume) in a sealed 100 ml glass bottle (Cygnar et al., 2010). Odorants were delivered via a picospritzer controlled by pCLAMP software (version 9.2, Molecular Devices) as a 100 ms pressurized pulse injected into a continuous stream of humidified air flowing over the tissue. Electrodes (1-3 MΩ) were made of borosilicate glass capillaries filled with 0.5% SeaPlaque agarose (Lonza) in modified Ringer's solution (135 mM NaCl, 5 mM KCl, 1 mM CaCl_2_, 1.5 mM MgCl_2_, 10 mM HEPES, pH 7.4). Responses to odor stimuli were recorded from turbinates II and IIb using a Multiclamp 700A amplifier controlled by pClamp software (version 9.2, Molecular Devices). EOG responses were measured as the maximal peak amplitude from the pre-pulse baseline using Clampfit software (Molecular Devices). Data were normalized to the mean response to pure AA in control mice. All data are expressed as normalized mean±s.e.m.

### BrdU assays

In P18 mice, the thymidine analog BrdU (Alfa Aesar, H27260) was administered via two i.p. injections, 2 h apart at a concentration of 50 mg/kg. The animals were sacrificed 2 h after the final BrdU injection to assess proliferation or sacrificed at 12 days or 25 days post-BrdU injection for the cellular birthdating experiments. We chose P18 as the age to inject the mice as it takes on average 10-12 days after basal cell division for a cell to become a mOSN (Liberia et al., 2019), and we aimed to birthdate the stem cells responsible for the shift in neuronal population observed at 1 month of age. Twenty images per mouse were collected with a 40× oil immersion optical lens using a Nikon A1 confocal microscope. The number of BrdU-labeled cells per mm of the OE was calculated as a measure of proliferation. BrdU birthdating experiments assessed the relative amount of BrdU/GAP43 double-labeled OSNs compared with BrdU/OMP dual-labeled cells per mouse.

### MMZ assay

One-month-old mice were administered an i.p. injection of 70 mg/kg of MMZ in sterilized PBS. MMZ solution was made fresh and protected from UV degradation. Mice were euthanized 1 month after MMZ injection and tissue was processed for histology, as described earlier.

### Western blot

The olfactory mucosa of each mouse was harvested after CO_2_ euthanasia. Samples were then homogenized in lysis buffer (250 μl of lysis buffer per 10 mg of tissue) using a mortar and pestle and centrifuged at 10,000 ***g*** for 10 min at 4°C. The total protein concentration was determined by a standard curve, utilizing a Bio-Rad DC Protein Assay (Bio-Rad, 500-0113) and spectrophotometer (NanoDrop, ND-1000). A solution of 120 μl of each sample with 1 μg/μl of protein, 1×SDS, and lysis buffer was prepared on ice. Sample solutions were then placed in 90°C for 2 min. Running buffer consisting of 1×SDS Running Buffer (Novex, NP0001), NuPage antioxidant (1:1000, Invitrogen, NP0005) in MilliQ ultra-pure water and NuPAGE Bis-Tris protein gel (Invitrogen, NP0335BOX) were added to the minitrans-blot cell (Bio-Rad, 1703989), and 15 μl of each sample and 10 μl of each protein ladder, Magic Mark (Invitrogen, LC5602) and SeeBlue Plus 2 (Invitrogen, LC5925) were added to the wells of the gel. Electrophoresis was run for 1 h at 195 V at T_rm_. Samples were then transferred onto a membrane at 100 V at T_rm_ for 1 h or at 40 V for 12-16 h at 4°C. Next, immunoblotting was conducted by blocking in TBSTM (1×TBS, 0.1% Tween 20, 5% milk powder) for 1 h at T_rm_. The membrane was incubated with primary antibody in TBSTM for 1 h at T_rm_, and rinsed four times with 1×TBST for 10 min on a shaker. The blot was incubated in diluted secondary antibody (1:1000) in TBSTM at T_rm_ for 1 h and then washed four times with 1×TBST for 10 min. The primary antibodies used were mouse anti-ARL13B (1:500, NeuroMab, 75-287) and the housekeeping protein rabbit anti-actin (1:2000, Sigma-Aldrich, A5060) and the secondary antibodies were HRP-conjugated goat anti-mouse (Invitrogen, 626520) and HRP-conjugated goat anti-rabbit (Invitrogen, 656120). Following incubation with antibodies, the membrane was incubated with enhanced luminal reagent and oxidizing reagents (Perkin Elmer, 50-904-9325) in a 1:1 ratio for 1 min. Finally, the blot was imaged using a bioimaging system (EpiChemi3 Darkroom, UVP BioImaging Systems, G53-BC-110403 95-0310-11). Immunoblotting with anti-ARL13B was performed first. After rinsing in 1×TBS on a shaker for 12-16 h at 4°C, the same blot was then stained with anti-actin the following day. During the analysis, the intensity of the ARL13B band (48 kDa) was normalized to the intensity of the actin band (42 kDa) of the sample and the adjusted ARL13B signal in *123-Arl13b* mice was then normalized to that of WT mice. All data are expressed as normalized mean±s.e.m.

### Image analysis

Images of immunostained tissue were collected on a Nikon A1 confocal microscope. All analysis of the OE involved imaging *z*-stacks at 0.5 μm thickness of five designated locations (septum, endoturbinates II and III, ectoturbinates 1 and 2′) for the left and the right nostril, for a total of ten images per mouse unless noted otherwise. All analysis of immunofluorescence images was carried out on *z*-projections of maximum intensity files, except for the assessment of ARL13B-positive cilia, for which we did not flatten the image. Images were processed and fluorescence intensity was quantified using ImageJ/Fiji software and assembled using PowerPoint (Microsoft). Cellular counts were normalized to the length of the OE in the image. Quantification of glomerular area and corrected total cell fluorescence for anti-TH immunostaining were calculated for each glomerulus using a modified version of this method (McCloy et al., 2014).

### Statistical analysis

Data are expressed as mean±s.e.m. and as percentage relative frequency. All statistical analyses and graph making were performed using GraphPad Prism 6 software. Student's *t*-test was used for comparison of the results between two groups. For data displayed as percentages, a Student's *t*-test was run after an arcsine transformation was performed on Excel (Microsoft) utilizing DEGREES{ASIN[SQRT(Cell/100)]} function. Statistical differences are given as *P<0.05, **P<0.01, ***P<0.001 and ****P<0.0001 compared with control or untreated groups, respectively.

## Supplementary Material

10.1242/develop.201116_sup1 Supplementary information
