# Supplementary material for: The role of a ciliary GTPase in the regulation of neuronal maturation of olfactory sensory neurons
Source: Development. 2023 Jan 19;150(2):dev201116. doi: 10.1242/dev.201116 (PMC10110495; doi:10.1242/dev.201116)
Supplement: Supplementary information [file develop-150-201116-s1.pdf]

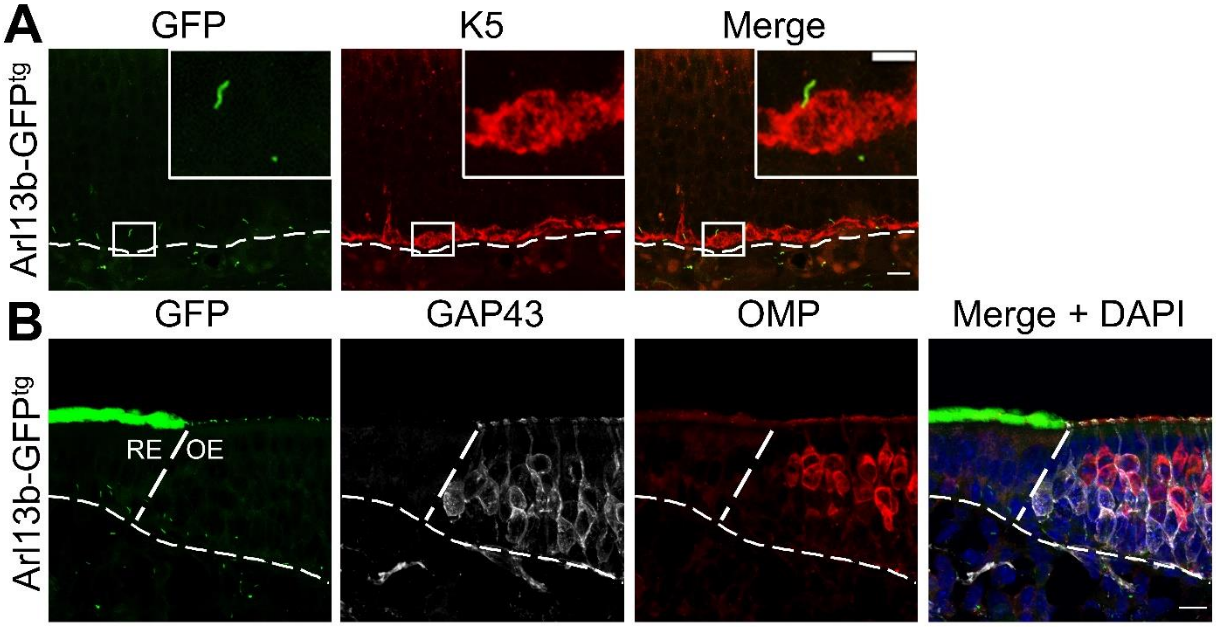

Fig. S1. ARL13B expression in the OE and the RE. (A) Representative confocal image of the nasal cavity of *Arl13b-GFP^{tg}* immunolabeled with K5, marking HBCs, showing ARL13B-GFP positive primary cilia emanating from HBCs. **(B)** Representative confocal image of a coronal section of the nasal cavity of *Arl13b-GFP^{tg}* mouse immunolabeled for GAP43 (iOSN), OMP (mOSNs), and DAPI (nuclei). The interface of the respiratory (RE) and olfactory epithelium (OE) as shown by the longer dashed lines. P21 mice. Shorter dashed lines, basement membrane. Scale bars: 5 µm **(inset)**,10 µm **(whole)**.

Development • Supplementary information

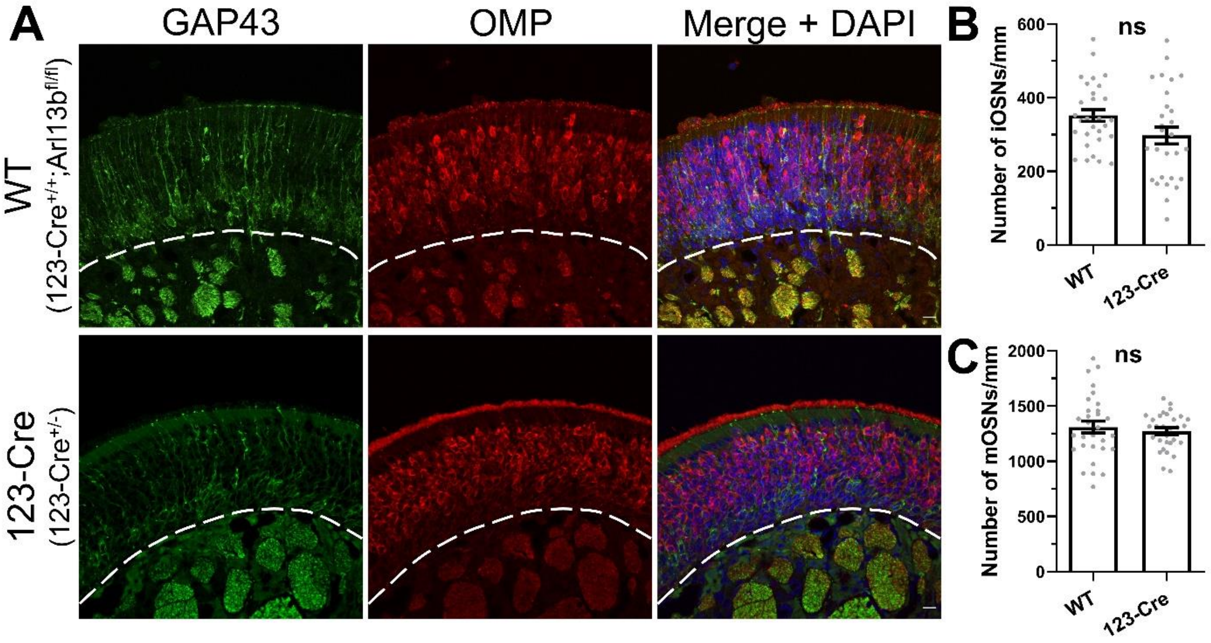

Fig. S2. Expression of *Cre* under the 123 promoter is not cytotoxic. (A) Immunofluorescent images of the OE of *123-Cre (123-Cre$^{+/-}$)* and WT (*123-Cre$^{+/+}$; Arl13b$^{fl/fl}$*) mice at 1m labeled with GAP43 for iOSNs and OMP for mOSNs. **(B)** Quantification of the number of iOSNs/mm of OE (WT, 352 ± 16.05 iOSNs/mm; *123-Cre*, 297.5 ± 22.91 iOSNs/mm). **(C)** Quantification of the number of mOSNs/mm of OE (WT, 1,310 ± 53.88 mOSNs/mm; *123-Cre*, 1272 ± 30.21 mOSNs/mm**).** Data for WT shown in **(B)** and **(C)** is the same as that of **Fig. 3D&E**, respectively and was referenced to compare to *123-Cre* mice. Dashed lines, basement membrane. Scale bar: 10 µm. N=3 mice per genotype. n.s. denotes non-significance (p>0.05), by unpaired two-tailed Student's *t*-tests. Values represent the mean ± s.e.m.

Development • Supplementary information

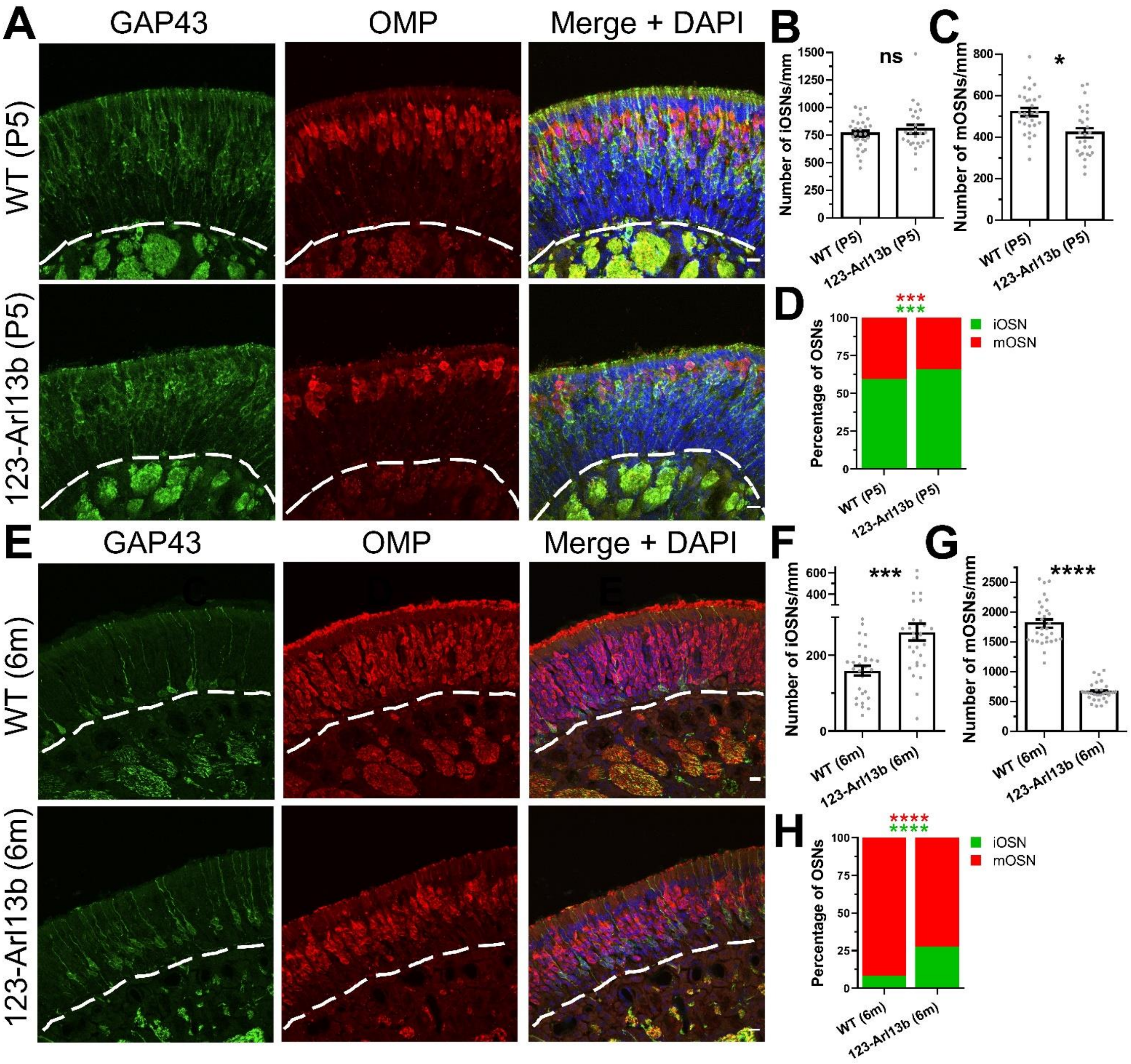

Fig. S3. Characterization of the OE in *123-Arl13b* mice at P5 and 6m. Immunofluorescent images of the OE of *123-Arl13b* and WT mice at P5 **(A)** and 6m **(E)** with analysis of the number of iOSNs **(B,F)** and mOSNs **(C,G)** along with the percentage of OSNs **(D, H)**. **(B)** Quantification of the number of iOSNs per mm of the OE of P5 *123-Arl13b* and WT mice (WT, 765.2 ± 26.12 iOSNs/mm; *123-Arl13b*, 805.9 ± 39.98 iOSNs/mm). **(C)** Quantification of the number of mOSNs per mm of the OE of P5 *123-Arl13b* and WT mice (WT, 521.3 ± 19.83 mOSNs/mm; *123-Arl13b*, 421.1 ± 22.85 mOSNs/mm). **(D)** Quantification of the ratio of iOSNs/mm to mOSNs/mm of P5 *123-Arl13b* and WT mice (WT, 59.4467/40.5533; *123-Arl13b*, 65.9083/34.0917). **(F)** Quantification of the number of

Development • Supplementary information

iOSNs per mm of the OE of 6m *123-Arl13b* and WT mice (WT, 159.5 ± 12.93 iOSNs/mm; *123-Arl13b*, 261.0 ± 22.06 iOSNs/mm). **(G)** Quantification of the number of mOSNs per mm of the OE of 6m *123-Arl13b* and WT mice (WT, 1809 ± 68.25 mOSNs/mm; *123-Arl13b*, 661.7 ± 27.55 mOSNs/mm). **(H)** Quantification of the ratio of iOSNs/mm to mOSNs/mm of 6m *123-Arl13b* and WT mice (WT, 8.30375/91.6963; *123-Arl13b*, 27.6268/72.3732). Dashed lines, basement membrane. Scale bar: 10 μm. N=3 mice per genotype for each age group. n.s. denotes non-significance ($p>0.05$), *$p<0.05$, ***$p<0.001$, ****$p<0.0001$, by unpaired two-tailed Student's *t*-tests. Values represent the mean ± s.e.m.

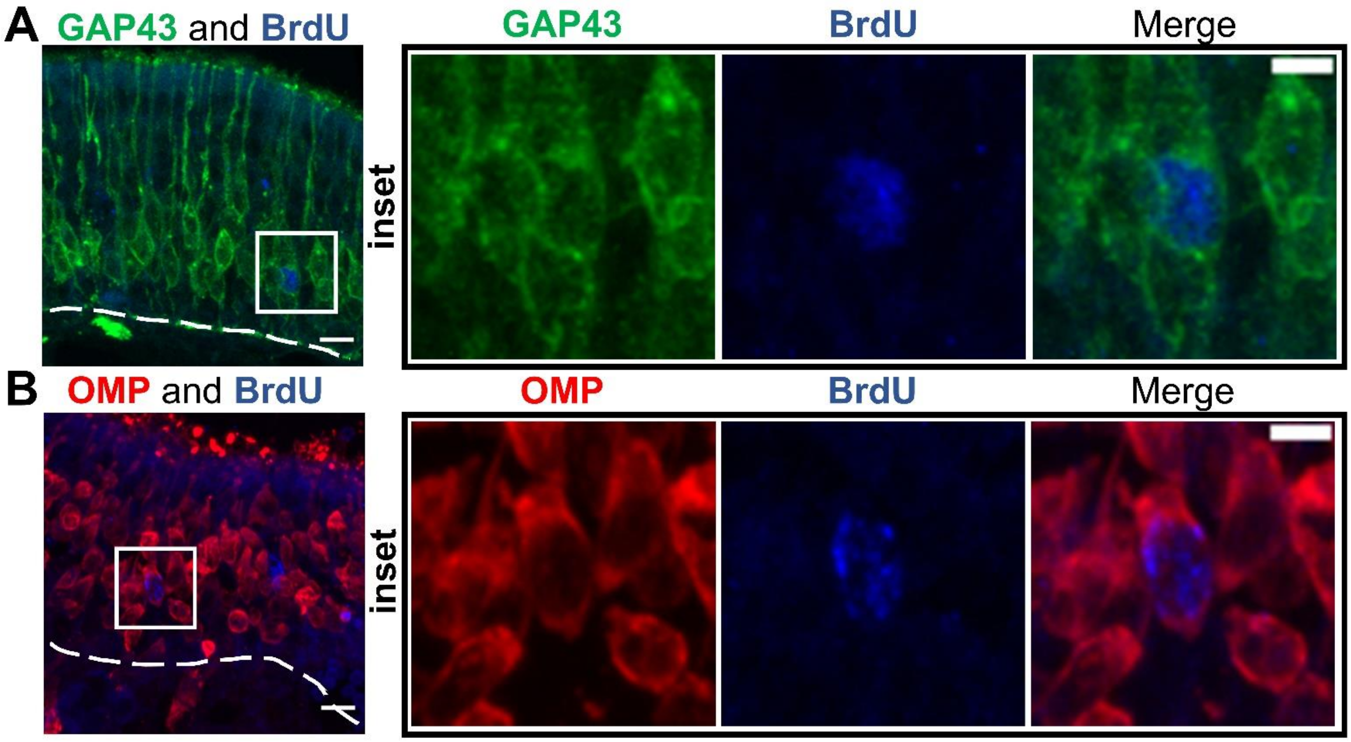

Fig. S4. Representative images of BrdU birth-dating experiments of OSNs in the olfactory system. Confocal image of the OE of BrdU injected mice, co-labeled for BrdU and either GAP43 for iOSNs **(A)** or OMP for mOSNs **(B)**. Insets show colocalization of neurons that are double positive for BrdU and either GAP43 or OMP. Dashed lines, basement membrane. Scale bars: 5 µm **(inset)** and 10 µm **(whole)**.

Development • Supplementary information

A

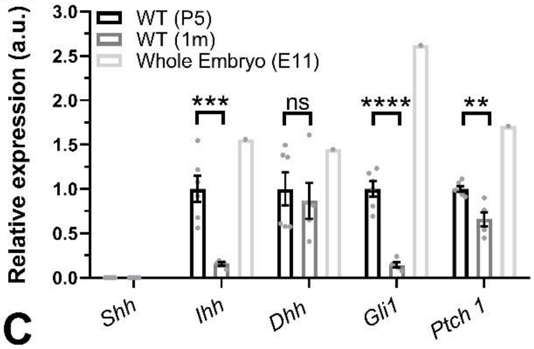

B

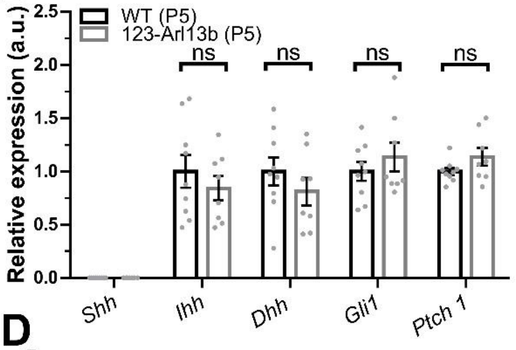

C

Condition	CT value for *Shh*	N of samples
WT (P5)	ND	9 mice
123-Arl13b (P5)	ND	8 mice
WT (1m)	ND	5 mice
123-Arl13b (1m)	ND	6 mice
Embryo (E11)	28.935±0.190	1 reference sample (run on 6 plates)

D

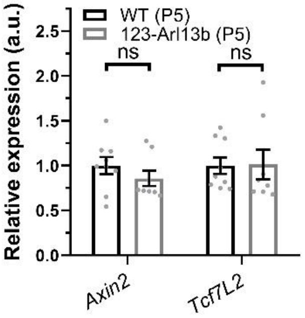

Fig. S5. Age related decline in HH signaling in the WT olfactory mucosa. RT-qPCR for *Shh*, *Ihh*, *Dhh*, *Gli1*, and *Ptch1* for **(A)** WT mice at P5 compared to 1m as well as **(B)** WT and *123-Arl13b* at P5. **(C)** Table showing that *Shh* transcript was not determined in any sample of the olfactory mucosa tested and the cycle threshold (CT) value in the E11 embryo positive control. **(D)** RT-qPCR of *Axin2* and *Tcf7L2* of P5 WT and *123-Arl13b* mice. N=6 WT, N=5 *123-Arl13b* mice **(A)**; N=9 WT, N=8 *123-Arl13b* mice **(B,D)**. ns for p>0.05, **p<0.01, ***p<0.001, ****p<0.0001, by unpaired two-tailed Student's *t*-tests. Values represent the mean ± s.e.m.

Development • Supplementary information

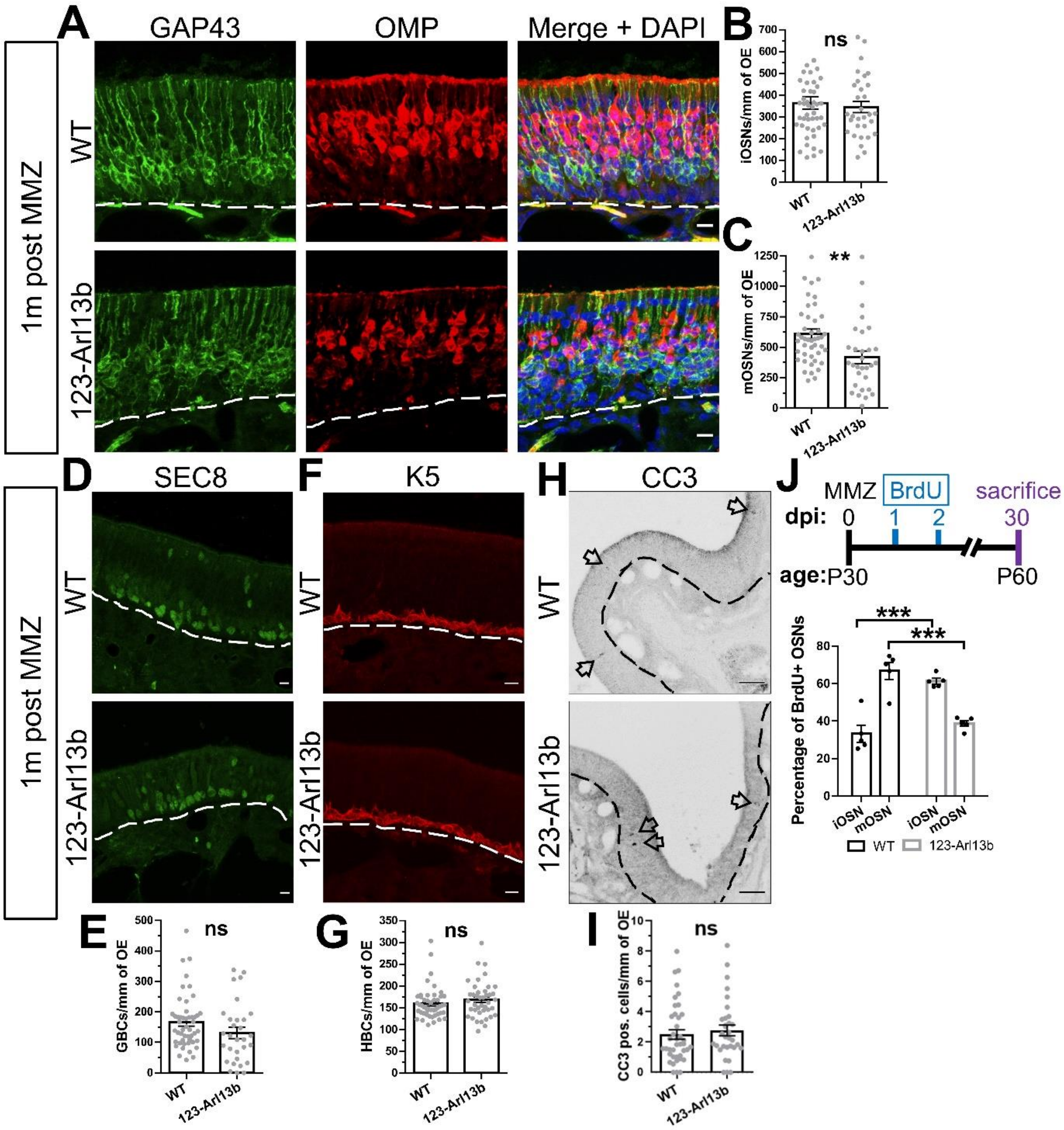

Fig. S6. Loss of ARL13b in iOSNs leads to a delay in maturation in the regenerating OE. (A) Representative image of the OE 1m after MMZ administration immunolabeled for iOSNs (GAP43) and mOSNs (OMP). **(B)** Quantification of the number of iOSNs per mm of the OE in regenerating *123-Arl13b* and WT mice (WT, 363.8 ± 29.21 iOSNs/mm; *123-Arl13b*, 344.6 ± 25.56 iOSNs/mm). **(C)** Quantification of the number of mOSNs per mm of the OE in regenerating *123-Arl13b* and WT mice (WT, 611.0 ± 38.33 mOSNs/mm; *123-Arl13b*, 418.2 ± 51.83 mOSNs/mm). **(D)** Representative image of the nasal cavity of the regenerating OE stained for GBCs (marked by SEC8). **(E)** Quantification of the number of GBCs per mm of the OE in regenerating *123-Arl13b* and WT mice (WT, 166.2 ± 12.47 GBCs/mm; *123-Arl13b*, 130.8 ± 18.09 GBCs/mm). **(F)** Representative images of the nasal cavity of the regenerating OE stained for HBCs (marked by K5). **(G)** Quantification of the number of HBCs per mm of the OE in regenerating *123-Arl13b* and WT mice (WT, 159.3 ± 5.167 HBCs/mm; *123-Arl13b*,

Development • Supplementary information

168.4 ± 6.489 HBCs/mm). **(H)** Representative image of the OE immunolabeled for CC3 marking apoptosis. **(I)** Quantification of the number of CC3+ cells per mm of the OE in regenerating *123-Arl13b* and WT mice (WT, 2.502 ± 0.3136 CC3+ cells/mm; *123-Arl13b*, 2.775 ± 0.3511 CC3+ cells/mm). **(J, top)** Diagram of experimental design where mice were injected with BrdU, 1dpi and 2dpi of MMZ and euthanized 1m after MMZ injection. **(J, top)** *123-Arl13b* mice showed an increase in the percentage of BrdU/GAP43 co-labeled neurons and a decrease in BrdU/OMP co-labeled neurons compared to those in WT (percentage of iOSNs/percentage of mOSNs: WT, 33.23/66.77 ± 4.578; *123-Arl13b*, 61.27/38.73 ± 1.491). Dashed lines, basement membrane. Scale bars: 10 μm **(A,D,F)** and 50 μm **(H)**. N=5 mice for both genotypes. ns for p>0.05, **p<0.01, ***p<0.001, by unpaired two-tailed Student's *t*-tests. Values represent the mean ± s.e.m.

Development • Supplementary information

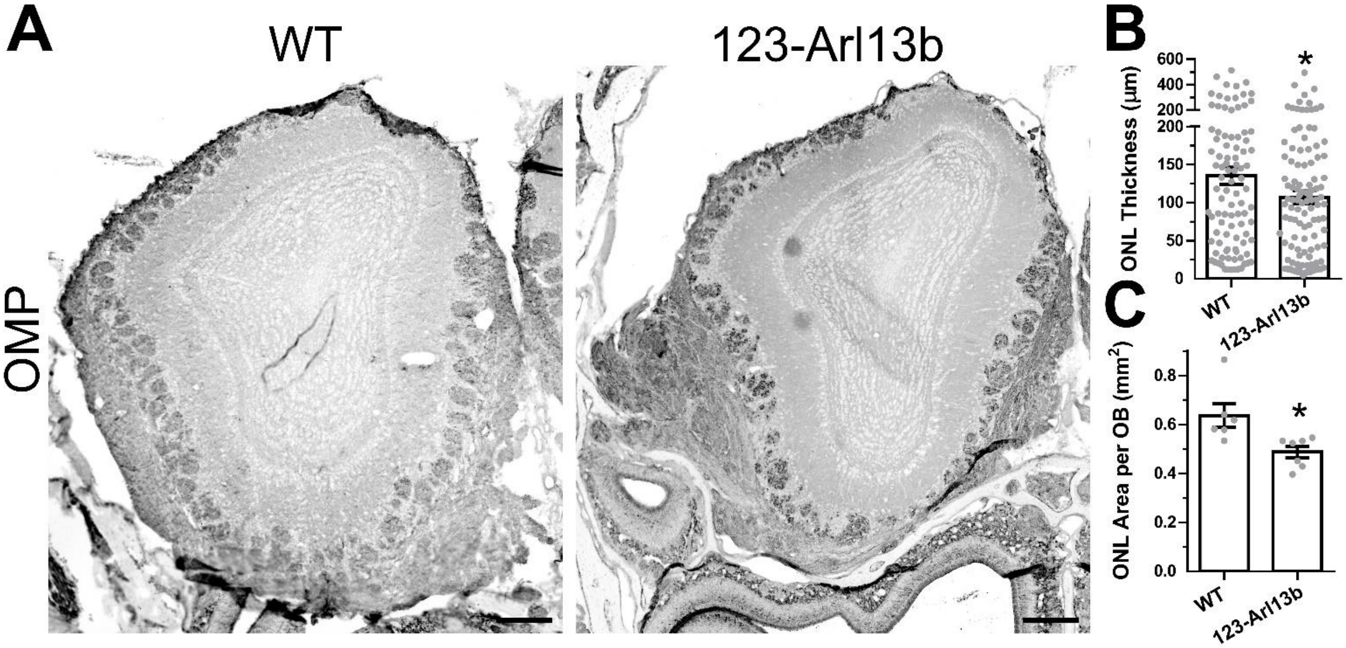

Fig. S7. Loss of Arl13b in iOSNs results in a smaller olfactory nerve layer. (A) Representative whole-stitch coronal section of the olfactory bulb immunolabeled for OMP, showing the olfactory nerve layer (ONL) as the outermost layer. **(B)** Quantification of the thickness of the ONL of WT and *123-Arl13b* mice throughout the ONL (WT, 135.4 ± 11.20 µm, N=4 mice; *123-Arl13b*, 107.2 ± 8.33 µm, N=4 mice). **(C)** Quantification of the area of the ONL of WT and *123-Arl13b* mice per OB (WT, 0.636 ± 0.0482 mm^2 , N=3 mice; 123-Arl13b, 0.489 ± 0.0228 mm^2, N=4 mice). Scale bars: 200 µm. *p<0.05, by unpaired two-sided Student's *t*-test. Values represented as mean ± s.e.m.

Development • Supplementary information
