# Supplementary material for: The role of a ciliary GTPase in the regulation of neuronal maturation of olfactory sensory neurons
Source: Development. 2023 Jan 19;150(2):dev201116. doi: 10.1242/dev.201116 (PMC10110495; doi:10.1242/dev.201116)
Supplement: Supplementary file 1 [file develop-150-201116_review_history.pdf]

The role of a ciliary GTPase in the regulation of neuronal maturation of olfactory sensory neurons

Julien C. Habif, Chao Xie, Carlos de Celis, Kirill Ukanov, Warren W. Green, Jordan C. Moretta, Lian Zhang, Robert J. Campbell and Jeffrey R. Martens
DOI: 10.1242/dev.201116

Editor: François Guillemot

Review timeline

Original submission:	9 August 2022
Editorial decision:	13 September 2022
First revision received:	13 December 2022
Accepted:	19 December 2022

Original submission

First decision letter

MS ID#: DEVELOP/2022/201116

MS TITLE: A novel role for a ciliary GTPase in the regulation of neuronal maturation of olfactory sensory neurons

AUTHORS: Julien C. Habif, Chao Xie, Carlos de Celis, Kirill Ukanov, Warren W. Green, Jordan C. Moretta, Lian Zhang, Robert J. Campbell, and Jeffrey R. Martens

I have now received the reports of two referees on your manuscript and I have reached a decision. The reports are appended below and you can access them online: please go to BenchPress and click on the 'Manuscripts with Decisions' queue in the Author Area.

As you will see, the referees express great interest in your work, but they also have significant criticisms and recommend a substantial revision of your manuscript before we can consider publication. If you are able to revise the manuscript along the lines suggested, which may involve further experiments, I will be happy to receive a revised version of the manuscript. You should in particular address the request of referee 1 that you provide additional mechanistic insights into the defects of Arl13b mutant mice, e.g. by investigating defects in Shh and Wnt signaling pathways. Your revised paper will be re-reviewed by the original referees, and its acceptance will depend on your addressing satisfactorily all their major concerns. Please also note that Development will normally permit only one round of major revision. If it would be helpful, you are welcome to contact us to discuss your revision in greater detail. Please send us a point-by-point response indicating your plans for addressing the referee’s comments, and we will look over this and provide further guidance.

Please attend to all of the reviewers' comments and ensure that you clearly highlight all changes made in the revised manuscript. Please avoid using 'Tracked changes' in Word files as these are lost in PDF conversion. I should be grateful if you would also provide a point-by-point response detailing how you have dealt with the points raised by the reviewers in the 'Response to Reviewers' box. If you do not agree with any of their criticisms or suggestions please explain clearly why this is so.

© 2023. Published by The Company of Biologists under the terms of the Creative Commons Attribution License (https://creativecommons.org/licenses/by/4.0/).

Reviewer 1

Advance summary and potential significance to field

The article by Julien Habif and coworkers provides a very interesting analysis of the ole of Arl13b and primary cilia in controlling the process of maturation and connectivity of the olfactory sensory neurons. The authors observed that ARL13b is expressed in primary cilia of immature OSNs but not in the sensory cilia of by mature OSNs. The authors show that conditional ablation of the functional form of Arl13b results in delayed OSNs' maturation, reduced olfactory performance, and in changes in size and number of the glomeruli in the main olfactory bulb. These observations are relevant and of broad interest.

The experiments are generally well designed. Images are of high quality and compelling. However, in the current form the work lacks mechanistic data.

Comments for the author

Major:

The results shown in the paper are very interesting, however in the present form the manuscript is a nice characterization of a mutant without a real investigation of the mechanisms. The beautiful data show us that Arl13b is important for olfactory maturation, but the authors do not explain us why. What does Arl13b do in the immature neurons? What signal is missing to facilitate the transition to mature? Is the maturation of the OSN controlled by extrinsic signals?

1) The phenotype of the Arl13b is an enrichment of immature neurons that are the neurons that express Arl13b. So, if Arl13b is needed for the transition to mature OSN the authors could do FACS sorting of immature neurons and bulk RNA seq or, better, Sc-seq comparison and see what genes are differentially regulated in the immature OSNs. This will give insights about what is going on and make this a very relevant paper.

2) The primary cilium is a "signaling antenna", that is right. So, are Wnt or Shh signaling altered in the Arl13b cKO? The authors could check if there are differences in Gli1 expression which is positively upregulated regulated by Shh.

For Wnt the authors could try to do immunostaining against stabilized b-Catenin. As a suggestion, the authors could consider trying anti stabilized β-Catenin (Ser33/37/Thr41) (D13A1) Rabbit mAb #8814. In our hands works 1:200 with and without antigen retrieval (DAB), it shows some nuclear staining the GBCs and early iOSN (not published). If that works, the authors could quantify nuclear staining and see if there are differences in Wnt signaling in the cKOs.

Investigation of the expression of some other known gene targets of Wnt, Shh via in IH or IHC should be included, that could provide important mechanistic information.

3) Cre can be toxic in mouse lines that express high levels of Cre. Quantifications of immature and mature OSNs of 123Cre+/- mice should be included.

4) Please note that T-test of percentage values cannot be done without Arcsine transformation of the data.

Minor

1) Figure 1, though quite convincing as it is, would benefit from a quantification showing the rate of GAP43 and ARL13B colocalization.

2) Line 104-106 should be rewritten I suggest writing "Next we generated an Arl13b conditional KO by crossing…. "

3) If the authors want to call the cKO "123-Arl13b" they should first write the correct genotype "123-Cre+/-/Arl13bfl/fl" and then state that they will call, throughout the article, mice with this

© 2023. Published by The Company of Biologists under the terms of the Creative Commons Attribution License (https://creativecommons.org/licenses/by/4.0/).

genetic makeup 123-Arl13b. Moreover, they should clearly state throughout the manuscript the genotype for all controls and mutants.

4) BrDU birth dating expreiments should not be called lineage tracing.

Reviewer 2

Advance summary and potential significance to field

Briefly, this focused study has studied the differentiation of immature olfactory sensory neurons (iOSNs) to a mature state (mOSNs) using an iOSN-specific Arl13b-null murine model (123-Arl13b). Based on the presented findings the authors suggest that ARL13B has a transient expression in primary cilium on iOSNs, which is lost in multiple long cilia of mOSNs. Furthermore, 123-Arl13b mice exhibit an increase in the number of iOSNs and a decrease in the number of mOSNs compared to WT mice, which appears to be due to a delay in neuronal maturation. In addition, the loss of Arl13b result in a deformation of glomerular shape and an attenuation of its innervation.

Overall, the manuscript from Martens research group reflects a good study design and the results are clearly presented in text, images and graphs. However, the text also includes several minor errors and inconsistencies in figure legends references to figures and main text that should have been noted by the authors before submission. One major concern is that the olfactory nerve has not been clearly studied in 123-Arl13b or wt mice, as well as the lack of discussion of any suggested functional role of ARL13B in other ciliated cells. If the below minor and major comments are addressed, this manuscript will be informative for both the olfactory and cilia research communities, as well as for researchers in the GTPase field, as it describes a novel role for the GTPase ARL13B, expressed in primary cilia in iOSNs, in the differentiation from iOSNs to mOSNs.

Comments for the author

Major comments
Introduction (and discussion):
1) Lines 44 and 260: The maturation process of OSNs have been reviewed by others than the corresponding author´s lab, and should be included as references. For example, Wang et al., 2017, Chem. Senses; Kurian et al., 2021, Cell Tissue Res; Dorrego-Rivas and Grubb, 2022, Open Biol.

Results/Figures:
2) Fig. 5G and S.Fig. 4F: Even if no change in the number of HBCs, characterized by K5 expression, has been observed, in Fig. 5G and S.Fig. 4F the K5 expression appears altered in 123-Arl13b mice compared to wt.
In Fig. 5G the K5 expression pattern appears decreased in 123-Arl13b mice compared to wt, and in S.Fig. 4F the opposite can be noted; that the K5 expression pattern appears increased in 123-Arl13b mice compared to wt. The authors should address this.

3) Lines 174-176. The authors need to explain why P18 mice and not P30 were used for the BrdU proliferation assay.

4) The authors show that neurogenesis is disturbed in 123-Arl13b mice, and that loss of Arl13b in iOSNs causes a deformation in glomerular shape and diminished OSN innervation of glomeruli. Therefore, the authors should also include measurements of the size (thickness) and potential other parameters of the olfactory nerve in 123-Arl13b compared to wt mice.

Discussion:
5) Lines 266-270. The authors should expand the discussion regarding AC3-/- mice in comparison to the 123-Arl13b mice. For example, include information that AC3-/- mice exhibit an increase in iOSNs, a reduction in mOSNs and increased apoptosis. They should also suggest an explanation (speculate) why odor detection is reduced in 123-Arl13b mice, but abolished in AC3-/- mice.

© 2023. Published by The Company of Biologists under the terms of the Creative Commons Attribution License (https://creativecommons.org/licenses/by/4.0/).

6) The authors should include a discussion section regarding the functional role of ARL13B in other ciliated cells, as well as the phenotype of potential other 123-Arl13b knock-out/down ciliated cells in comparison with loss of 123-Arl13b in iOSNs. This would be informative.

7) Lines 271-272. The conclusion: "ARL13B's role on OSN maturation may be through effects on the olfactory signaling transduction cascade or other signaling mechanisms" does not fit with this discussion section. Please revise.

Minor comments.

Throughout the manuscript:
8) Make sure to explain all abbreviations when first used.

9) Make sure that all figures are referred to in the text and that references to figures are correct (including that figure references should be positioned after sentences that actually presents the results, not after describing the experiment).

10) Since the study includes many different ages of mice it would be helpful if the analyzed ages consistently are stated in the figure legends. Also, the authors should decide whether to use the term P30 or 1m to indicate one month old mice.

Results:
11) Results related to Fig. 1E. The authors should clarify whether all GAP43+ iOSNs possess an ARL13B+ primary cilium, or state the percentage.

12) Lines 130-131. The conclusion should be more precise than just stating "… caused a shift in neuronal population".

13) Lines 152-155. This part would be easier to follow if the authors explain the average days from basal cell division to a mOSNs before they describe the BrdU injection protocol.

Figures:
14) Figs. 1A-C would be more informative if inserts, similar to those in Fig. 1E, were included.

Discussion:
15) Lines 288-289. The sentence "Additionally, HH signaling was important for the maturation of enteric neural crest cells from human pluripotent stem cells (Lau et al., 2019)" should be removed, as it is not related to ARL13B or ciliated cells.

First revision

Author response to reviewers' comments

Point-by-point response to the reviewers' comments

We are pleased to submit a revised manuscript for your consideration. We have included additional data, tested a suggested antibody, made the appropriate adjustments to the text, and expanded upon our discussion. Overall, we feel that the manuscript has greatly improved thanks to the thoughtful comments of the Reviewers. Please find our point-by-point response to the Reviewers' comments below. Thank you for your consideration.

Reviewer 1 Comments

Major comments

The results shown in the paper are very interesting, however in the present form the manuscript is a nice characterization of a mutant without a real investigation of the mechanisms. The beautiful

© 2023. Published by The Company of Biologists under the terms of the Creative Commons Attribution License (https://creativecommons.org/licenses/by/4.0/).

data show us that Arl13b is important for olfactory maturation, but the authors do not explain us why.

- What does Arl13b do in the immature neurons?
- What signal is missing to facilitate the transition to mature?
- Is the maturation of the OSN controlled by extrinsic signals?

1) The phenotype of the Arl13b is an enrichment of immature neurons that are the neurons that express Arl13b. So, if Arl13b is needed for the transition to mature OSN the authors could do FACS sorting of immature neurons and bulk RNA seq or, better, Sc-seq comparison and see what genes are differentially regulated in the immature OSNs. This will give insights about what is going on and make this a very relevant paper.

We thank the Reviewer for their suggestion. This study found that the GTPase ARL13B localized to primary cilia, a signaling hub, and that its loss in iOSNs disrupted maturation. Through phenotypic similarities and the known roles of ARL13B, we identified and, in the discussion, considered various signaling mechanisms that may have a role in OSN maturation, namely Wnt and HH. As the Reviewer suggested, we tested if these two signaling pathways were altered in the *123-Arl13b* mouse model (please see next comment). Interestingly, we found no changes in the gene expression of the readouts of either Wnt or HH. The Reviewer provided another way to identify signaling pathways that may be involved in ARL13B-mediated neuronal maturation which is to conduct RNA-sequencing. We agree that these are potentially very fruitful experiments that could generate new hypotheses. These experiments are very detailed and if any differences are identified would require validation. We aim to conduct RNA-sequencing in the future.

2) The primary cilium is a “signaling antenna”, that is right. So, are Wnt or Shh signaling altered in the Arl13b cKO? The authors could check if there are differences in Gli1 expression which is positively upregulated regulated by Shh.

For Wnt the authors could try to do immunostaining against stabilized b-Catenin. As a suggestion, the authors could consider trying anti stabilized ß-Catenin (Ser33/37/Thr41) (D13A1) Rabbit mAb #8814. In our hands works 1:200 with and without antigen retrieval (DAB), it shows some nuclear staining the GBCs and early iOSN (not published). If that works, the authors could quantify nuclear staining and see if there are differences in Wnt signaling in the cKOs.

Investigation of the expression of some other known gene targets of Wnt, Shh via in IH or IHC should be included, that could provide important mechanistic information.

We are grateful for these suggestions and have conducted various experiments to address this comment. Specifically, we tested if gene expression of HH and Wnt signaling were altered in the *123-Arl13b* mouse model compared to WT with RT-qPCR of whole lysate olfactory mucosa (Fig. 5, Fig. S5). As the Reviewer suggested Wnt and HH are the two most likely candidates which we highlighted in the manuscript. Utilizing RT-qPCR we discovered that there are in fact no alterations in gene expression of these pathways after loss of ARL13B in iOSNs. These findings were intriguing given the strong association of ARL13B with HH signaling. Additionally, we also discovered that some but not all of the HH ligands are present in the olfactory mucosa and we are the first to show an age dependent decline in expression of HH signaling in the OE (P5 compared to 1m).

<u>Our description of this experiment in the Results section is as follows [Lines 178-198]:</u>
Primary cilia are specialized organelles that are essential sites for signal transduction of many pathways, including Hedgehog (HH) and Wnt signaling (Wheway et al., 2018). In various cell types, loss of ARL13B results in low level constitutive activation of HH (Caspary et al., 2007, Larkins et al., 2011, Ferent et al., 2019, Shao et al., 2017). Therefore, we tested gene expression of HH ligands (Sonic HH (*Shh*), Indian HH (*Ihh*), and Desert HH (*Dhh*)) and readouts of the pathway (*Gli1*, *Ptch1*) in the olfactory mucosa of *123-Arl13b* mice. *Shh* transcript was not determined (cycle threshold (CT) > 38) at P5 and 1m in either genotype **(Fig. 5A, Fig. S5B)**, which corroborates earlier findings in P2 WT mice (Gong et al., 2009). We confirm that the previously published (Kopinke et al., 2017) *Shh* primer used was functional as we detected high levels of *Shh* in an E11 embryo control **(Fig. S5C)** as expected (Jiang and Hui, 2008, Krauss et al., 1993). Interestingly, we observed detectable levels of *Ihh* and *Dhh* in both P5 and 1m mice but no differences between WT and *123-Arl13b* mice **(Fig. S5A,**

© 2023. Published by The Company of Biologists under the terms of the Creative Commons Attribution License (https://creativecommons.org/licenses/by/4.0/).

Fig. 5A). Additionally, at 1m we determined that there was no difference between genotypes in the gene expression of the transcriptional targets of HH, *Ptch1* and *Gli* **(Fig. 5B)**. We repeated the assay in P5 mice where the number of iOSNs is the same between WT and *123-Arl13b* mice and corroborated the lack of change in HH signaling gene expression **(Fig. S5B)**. Interestingly, we found that expression of *Ihh*, *Ptch1*, and *Gli1* dramatically decreased in WT mice from P5 to 1m **(Fig. S5A)**. We next tested if there were any changes in Wnt signaling as it has been suggested to play a role in OSN maturation (Wang et al., 2011). However, at either P5 or 1m there was no difference in gene expression of the Wnt pathway readouts, *Axin2* and *Tcfl2* between WT and *123-Arl13b* mice **(Fig. S5D, Fig. 5C)**. Given the lack of difference in HH and Wnt gene expression between genotypes, these data suggest that the phenotypes observed in the *123-Arl13b* murine model are through a HH- and Wnt-independent mechanism.

Regarding, the suggestion of using the Beta Catenin antibody, we initially validated the antibody in HeLa cells as per the manufacturer's published results (see panel A below). We then tested the antibody in the nasal cavity at different concentrations, fixation, and with/without antigen retrieval (panel B). The antibody suggested is against non-phosphorylated (active) Beta Catenin which should be seen as nuclear staining (as seen in the HeLa cells), however we observe a high signal to noise ratio and signal if real that is not confined to the nucleus. Therefore, we did not feel comfortable using this antibody in the updated manuscript. We believe that the RT-qPCR data collected, addresses if ARL13B's role in immature OSNs is through Wnt signaling.

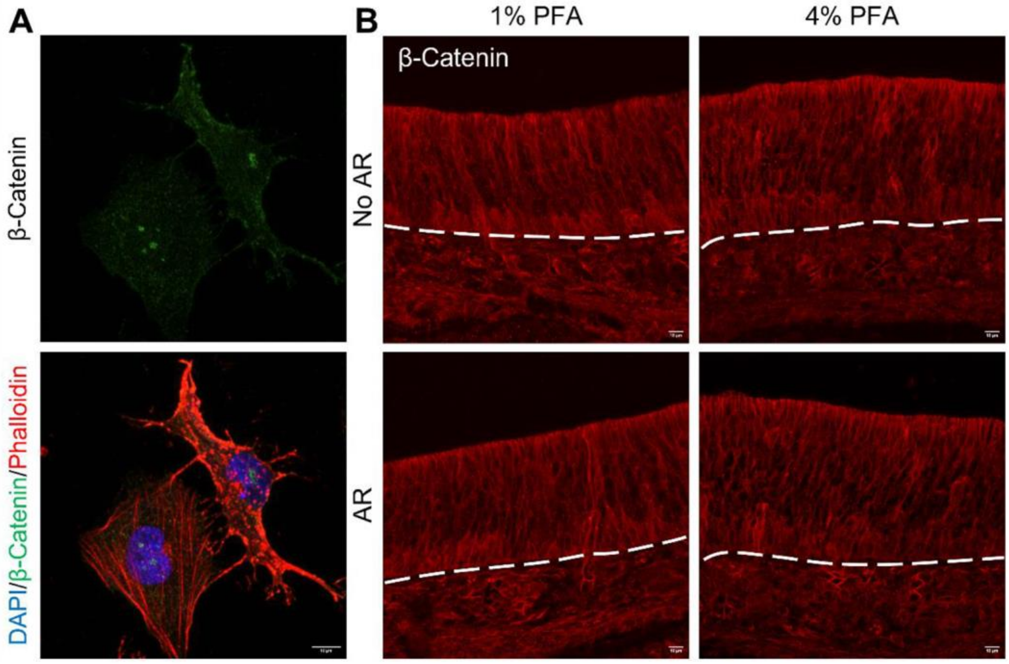

Staining for anti-ß Catenin antibody in Hela cells and the OE. (A) Hela cells immunolabeled for ß-Catenin and merged with DAPI and phalloidin. ß-Catenin signal is present in dense puncta in the nucleus, as expected. **(B)** The OE of P5 mice stained for ß-Catenin that were drop fixed in 1% PFA or 4% PFA and having either undergone antigen retrieval (AR) or not. No matter the condition there is a low signal to noise ratio and no clear ß-Catenin signal in the nuclei of the stem cells as expected (Wang et al., 2015). White line is basement membrane.

3) Cre can be toxic in mouse lines that express high levels of Cre. Quantifications of immature and mature OSNs of 123Cre+/- mice should be included.

We thank the reviewer for their comment. We assessed the neuronal composition of the OE in *123-Cre*$^{+/-}$ mice and compared it to that of WT mice (*123-Cre*$^{+/+}$;*Arl13b*$^{fl/fl}$). Please see Fig. S2.

Our description of this experiment in the Results section is as follows [Lines 139-142]:

© 2023. Published by The Company of Biologists under the terms of the Creative Commons Attribution License (https://creativecommons.org/licenses/by/4.0/).

Finally, when comparing 1m WT (*123-Cre*$^{+/+}$;*Arl13b*$^{fl/fl}$) and *123-Cre* (*123-Cre*$^{+/-}$) mice there was no difference in either the number of iOSNs or mOSNs **(Fig. S2)**, thus suggesting that the change in OE neuronal composition was not a cytotoxic effect from Cre Recombinase expression.

4) Please note that T-test of percentage values cannot be done without Arcsine transformation of the data.

We thank the reviewer for bringing this to our attention. We have performed an Arcsine transformation of the data and then ran a Student's *t*-test on the percentage values (Fig. 4). Additionally, we detail how this was performed in the updated Materials and Methods section.

Minor comments

1) Figure 1, though quite convincing as it is, would benefit from a quantification showing the rate of GAP43 and ARL13B colocalization.

The reviewer provided a great suggestion. We quantified the percentage of iOSNs (GAP43+) with ARL13B positive primary cilia in 1m mice. Please see Fig. 1F.

Our description of this experiment in the Results section is as follows [line 107-108]:
We determined that about 73% of iOSNs possess an ARL13B positive primary cilium in 1m WT mice **(Fig. 1F)**.

2) Line 104-106 should be rewritten I suggest writing "Next we generated an Arl13b conditional KO by crossing…. "

We thank the reviewer for their comment and have rewritten the statement as suggested.

3) If the authors want to call the cKO "123-Arl13b" they should first write the correct genotype "123-Cre+/- /Arl13bfl/fl" and then state that they will call, throughout the article, mice with this genetic makeup 123-Arl13b. Moreover, they should clearly state throughout the manuscript the genotype for all controls and mutants.

When first introducing the mouse model and in the materials and methods section we have now included a less ambiguous description of the genotypes and afterwards refer to the mouse model as *123-Arl13b*.

Our description of the genotype in the Results section is as follows [Lines 112-115]:
Next we generated an *Arl13b* conditional knock-out (cKO) mouse line by crossing *Arl13b*$^{fl/fl}$ mice (Su et al., 2012) with *123-Cre* mice (Hirata et al., 2006, Kaneko-Goto et al., 2013) **(Fig. 2A)**. For the following study, the WT mice were genotypically *123-Cre*$^{+/+}$;*Arl13b*$^{fl/fl}$ and the cKO mice referred to as *123-Arl13b* mice were *123-Cre*$^{+/-}$;*Arl13b*$^{\Delta/\Delta}$.

4) BrDU birth dating expreiments should not be called lineage tracing.

We have corrected the way we described the BrdU birth dating experiments and no longer refer to it as lineage tracing.

Reviewer 2 Comments

Major comments

Introduction (and discussion):
1) Lines 44 and 260: The maturation process of OSNs have been reviewed by others than the corresponding author´s lab, and should be included as references. For example, Wang et al., 2017, Chem. Senses; Kurian et al., 2021, Cell Tissue Res; Dorrego-Rivas and Grubb, 2022, Open Biol.

We apologize for not including more references related to OSN maturation. We have included the publications suggested to us by the reviewer along with a few others: (Kondo et al., 2010; Nickel et al., 2012; Rodriguez-Gil et al., 2015; Coleman et al., 2017; Fletcher et al. 2017)

© 2023. Published by The Company of Biologists under the terms of the Creative Commons Attribution License (https://creativecommons.org/licenses/by/4.0/).

Results/Figures:
2) Fig. 5G and S.Fig. 4F: Even if no change in the number of HBCs, characterized by K5 expression, has been observed, in Fig. 5G and S.Fig. 4F the K5 expression appears altered in 123-Arl13b mice compared to wt. In Fig. 5G the K5 expression pattern appears decreased in 123-Arl13b mice compared to wt, and in S.Fig. 4F the opposite can be noted; that the K5 expression pattern appears increased in 123-Arl13b mice compared to wt. The authors should address this.
We thank the reviewer for their observation. We do not observe difference in the expression pattern of K5 between WT and *123-Arl13b* mice either during homeostasis (Fig. 5G) nor regeneration (S. Fig. 4F). There is variability in the size and morphology of HBCs (K5+ cells) that we observe in both genotypes. Therefore, we have chosen representative images that demonstrate the similar K5 expression pattern between genotypes. See revised Fig. 6G and Fig. S6F.

3) Lines 174-176. The authors need to explain why P18 mice and not P30 were used for the BrdU proliferation assay.

We wanted to assay proliferation of the stem cells that would have produced the low amount of mOSNs and high amount of iOSNs observed in 1m old mice. Since it takes on average 10-12 days from basal cell division for an OSN to mature (Liberia et al., 2019), we therefore chose P18 (18+12=30 days). Additionally, we wanted to test if the increase in proliferation at P18 remained in 1m old mice, an age we test widely in the paper. Both time points consistently showed an increase in proliferation in *123-Arl13b* mice. We apologize for the confusion and have clarified this point in the revised manuscript.

Our description of this experiment in the Materials and Methods section is as follows [Lines 499-501]:
We chose P18 as the age to inject the mice as it takes on average 10-12 days after basal cell division for a cell to become a mOSN (Liberia et al., 2019), and we aimed to birthdate the stem cells responsible for the shift in neuronal population observed at 1m.

4) The authors show that neurogenesis is disturbed in 123-Arl13b mice, and that loss of Arl13b in iOSNs causes a deformation in glomerular shape and diminished OSN innervation of glomeruli. Therefore, the authors should also include measurements of the size (thickness) and potential other parameters of the olfactory nerve in 123-Arl13b compared to wt mice.

We thank the reviewer and agree with the recommendation. Analysis of the total area of the olfactory nerve layer (ONL) per OB and average thickness of the ONL have been quantified and added to Fig. S7.

Our description of this experiment in the Results section is as follows [Lines 247-250]:
The axons of OSNs fasciculate as they traverse from the periphery and form the outermost layer of the OB, known as the olfactory nerve layer (ONL). We stained coronal sections of the OB for OMP. We measured a statistically significant decrease in ONL thickness and area in 1m *123-Arl13b* mice compared to WT **(Fig. S7)**, which may be reflective of the loss of mOSNs.

Discussion:
5) Lines 266-270. The authors should expand the discussion regarding AC3-/- mice in comparison to the 123-Arl13b mice. For example, include information that AC3-/- mice exhibit an increase in iOSNs, a reduction in mOSNs and increased apoptosis. They should also suggest an explanation (speculate) why odor detection is reduced in 123-Arl13b mice, but abolished in AC3-/- mice.

The question from the reviewer was insightful. We have expanded the *123-Arl13b* and $AC3^{-/-}$ comparison in the discussion.

Our revised description of those findings in the Discussion section is as follows [Lines 303-313]:
There were various phenotypic similarities between $AC3^{-/-}$ and *123-Arl13b* mice including OE cellular composition (increase in iOSNs and decrease in mOSNs) and increased cell death. Odor detection in *123-Arl13b* mice was reduced but not abolished like in the $AC3^{-/-}$ mice. This was presumably due to loss of overall OSN ciliation in *123-Arl13b* mice while in the $AC3^{-/-}$ mice a critical component of the olfactory signalling transduction pathway was knocked out. Regardless, both

© 2023. Published by The Company of Biologists under the terms of the Creative Commons Attribution License (https://creativecommons.org/licenses/by/4.0/).

murine models resulted in loss of sensory input, albeit more severely in AC3$^{-/-}$ mice. It may therefore be hypothesized that both ARL13B and AC3's impact on maturation was resultant from sensory deprivation. Additionally, the critical process of synaptogenesis occurs during OSN maturation and recently the exuberant and plastic nature of the iOSN synapse has been revealed (Cheetham et al., 2016, Huang et al., 2022, Marcucci et al., 2011). Perhaps both the peripheral odorant detection and the subsequent synaptic transmission are necessary for the maturation of an OSN.

6) The authors should include a discussion section regarding the functional role of ARL13B in other ciliated cells, as well as the phenotype of potential other 123-Arl13b knock-out/down ciliated cells in comparison with loss of 123-Arl13b in iOSNs. This would be informative.

We thank the Reviewer for their comment. We have outlined the various parts of the discussion where we discuss the similarities and differences in the phenotypes between *123-Arl13b* mice and other ARL13B KO/knock down models.

Discussion [Lines 284-287]
Intriguingly, the loss of ARL13B in iOSNs resulted in mOSNs with shorter and fewer cilia compared to WT mOSNs. Our data support the documented role of ARL13B on cilia length (Caspary et al., 2007, Higginbotham et al., 2012, Thorpe et al., 2017) and ciliation (Hori et al., 2008, Larkins et al., 2011).

Discussion [Lines 320-323]
Also, the mutation of ARL13B caused a loss of WNT ligand in the neural tube (Horner and Caspary, 2011, Caspary et al., 2007). However, we observed no changes in gene expression of Wnt signaling in the *123-Arl13b* mouse model, suggesting that the maturation of OSNs regulated by ARL13B is not through the Wnt signalling pathway.

Discussion [Lines 333-337 and 341-343]
Interestingly, even though loss of *Arl13b* in various cell types resulted in a decrease of *Gli* transcriptional activation (Caspary et al., 2007, Su et al., 2012, Bay et al., 2018), we determined no difference in gene expression of either the HH ligands (no *Shh* expression, *Dhh*, and *Ihh*) or HH activity (*Gli1* and *Ptch1*) between WT and *123-Arl13b* mice suggesting that HH signaling pathway is not involved in the maturation of OSNs regulated by ARL13B.

We surmise that the phenotypes observed after loss of *Arl13b* in iOSNs are likely not through the canonical HH pathway, which is divergent from the numerous reports of ARL13B's regulation of HH signaling.

7) Lines 271-272. The conclusion: "ARL13B's role on OSN maturation may be through effects on the olfactory signaling transduction cascade or other signaling mechanisms" does not fit with this discussion section. Please revise.

We have removed this statement.

Minor comments.

Throughout the manuscript:
8) Make sure to explain all abbreviations when first used.

We thank the reviewer for bringing this to our attention. We have made sure to define every abbreviation when first used.

9) Make sure that all figures are referred to in the text and that references to figures are correct (including that figure references should be positioned after sentences that actually presents the results, not after describing the experiment).

We apologize for the error and have made the corresponding changes.

© 2023. Published by The Company of Biologists under the terms of the Creative Commons Attribution License (https://creativecommons.org/licenses/by/4.0/).

10) Since the study includes many different ages of mice it would be helpful if the analyzed ages consistently are stated in the figure legends. Also, the authors should decide whether to use the term P30 or 1m to indicate one month old mice.

The figure legends now include the ages of all of the mice used. We agree with this comment and now consistently utilize the nomenclature 1m instead of P30.

Results:

11) Results related to Fig. 1E. The authors should clarify whether all GAP43+ iOSNs possess an ARL13B+ primary cilium, or state the percentage.

We agree with the Reviewer's suggestion and have completed this experiment. Please see Fig. 1F.

Our description of this experiment in the Results section is as follows [line 107-108]:
We determined that about 73% of iOSNs possess an ARL13B positive primary cilium in 1m WT mice **(Fig. 1F)**.

12) Lines 130-131. The conclusion should be more precise than just stating "… caused a shift in neuronal population".

We agree with the reviewer and have modified the sentence.

Our description of this experiment in the Results section is as follows [line 142-143]:
These data indicate that the excision of Arl13b in iOSNs in 1m mice caused a shift in neuronal population, with increased iOSNs and a loss of mOSNs.

13) Lines 152-155. This part would be easier to follow if the authors explain the average days from basal cell division to a mOSNs before they describe the BrdU injection protocol.

We thank the reviewer for the advice and have made the appropriate modifications.

Figures:

14) Figs. 1A-C would be more informative if inserts, similar to those in Fig. 1E, were included.

We thank the reviewer for the suggestion. Our objective was to broadly show the localization of ARL13B at the apical surface in the OE relative to iOSNs and mOSNs. With our objective in mind, the resolution of Figs. 1A-C are not optimal to show and focus on the primary cilia. Regarding the aforementioned Fig. 1E, that shows endogenous ARL13B staining, these images were taken with a higher magnification objective and higher resolution (pixel dwelling time, etc). We believe that Fig. 1E (ARL13B staining) achieves the goal of this suggestion.

Discussion:

15) Lines 288-289. The sentence "Additionally, HH signaling was important for the maturation of enteric neural crest cells from human pluripotent stem cells (Lau et al., 2019)" should be removed, as it is not related to ARL13B or ciliated cells.

We thank the reviewer for their comment and have removed this sentence.

© 2023. Published by The Company of Biologists under the terms of the Creative Commons Attribution License (https://creativecommons.org/licenses/by/4.0/).

Second decision letter

MS ID#: DEVELOP/2022/201116

MS TITLE: A novel role for a ciliary GTPase in the regulation of neuronal maturation of olfactory sensory neurons

AUTHORS: Julien C. Habif, Chao Xie, Carlos de Celis, Kirill Ukanov, Warren W. Green, Jordan C. Moretta, Lian Zhang, Robert J. Campbell, and Jeffrey R. Martens
ARTICLE TYPE: Research Article

I am delighted to tell you that your manuscript has been accepted for publication in Development, pending our standard ethics checks.

Reviewer 1

Advance summary and potential significance to field

The Authors have addressed all of my concerns with the original manuscript. The revised manuscript is ready for publication.

Paolo E. Forni

Comments for the author

I have only one minor comment. In most of the article you use signaling with one "L", however, in few sentences you sue Signalling with two Ls, choose one or the other.

Reviewer 2

Advance summary and potential significance to field

This manuscript will be informative for both the olfactory and cilia research communities, as well as for researchers in the GTPase field, as it describes a novel role for the GTPase ARL13B, expressed in primary cilia in iOSNs, in the differentiation from iOSNs to mOSNs.

Comments for the author

The author has improved the manuscript significantly and addressed all my comments. With these clarifications I have no further comments on this solid and well-presented manuscript.

© 2023. Published by The Company of Biologists under the terms of the Creative Commons Attribution License (https://creativecommons.org/licenses/by/4.0/).
